# High-throughput stability screening for detergent-solubilized membrane proteins

**DOI:** 10.1038/s41598-019-46686-8

**Published:** 2019-07-17

**Authors:** Vadim Kotov, Kim Bartels, Katharina Veith, Inokentijs Josts, Udaya K. Tiruttani Subhramanyam, Christian Günther, Jörg Labahn, Thomas C. Marlovits, Isabel Moraes, Henning Tidow, Christian Löw, Maria M. Garcia-Alai

**Affiliations:** 1 Centre for Structural Systems Biology (CSSB), Notkestrasse 85, D-22607 Hamburg, Germany; 2 University Medical Center Hamburg-Eppendorf (UKE), Institute for Structural and Systems Biology, Notkestrasse 85, D-22607 Hamburg, Germany; 3 German Electron Synchrotron Centre (DESY), Notkestrasse 85, D-22607 Hamburg, Germany; 4 European Molecular Biology Laboratory Hamburg, Notkestrasse 85, D-22607 Hamburg, Germany; 5 The Hamburg Centre for Ultrafast Imaging (CUI) and Department of Chemistry, University of Hamburg, Martin-Luther-King-Platz 6, D-20146 Hamburg, Germany; 6 Research Centre Jülich, Institute of Complex Systems (ICS-6), Wilhelm-Johnen-Straße, D-52425 Juelich, Germany; 7 National Physical Laboratory, Hampton Road, Teddington, TW11 0LW UK; 8 Research Complex at Harwell, Rutherford Appleton Laboratory, Harwell Science and Innovation Campus, Didcot, OX11 0FA UK; 9 Department of Medical Biochemistry and Biophysics, Karolinska Institutet, Scheeles väg 2, SE-17177 Stockholm, Sweden

**Keywords:** Molecular biophysics, Membrane proteins

## Abstract

Protein stability in detergent or membrane-like environments is the bottleneck for structural studies on integral membrane proteins (IMP). Irrespective of the method to study the structure of an IMP, detergent solubilization from the membrane is usually the first step in the workflow. Here, we establish a simple, high-throughput screening method to identify optimal detergent conditions for membrane protein stabilization. We apply differential scanning fluorimetry in combination with scattering upon thermal denaturation to study the unfolding of integral membrane proteins. Nine different prokaryotic and eukaryotic membrane proteins were used as test cases to benchmark our detergent screening method. Our results show that it is possible to measure the stability and solubility of IMPs by diluting them from their initial solubilization condition into different detergents. We were able to identify groups of detergents with characteristic stabilization and destabilization effects for selected targets. We further show that fos-choline and PEG family detergents may lead to membrane protein destabilization and unfolding. Finally, we determined thenmodynamic parameters that are important indicators of IMP stability. The described protocol allows the identification of conditions that are suitable for downstream handling of membrane proteins during purification.

## Introduction

Numerous studies have addressed the gap between the large number of sequences representing integral membrane proteins (IMPs) in sequenced genomes (up to 30 percent)^1^ and the 863 unique known membrane protein structures reported up-to-date (http://blanco.biomol.uci.edu/mpstruc/). This highlights the difficulties along the path to structure determination such as low expression levels, solubilization from the native lipid bilayer and stability in a new membrane mimetic^2–4^. Most of these known unique structures were determined by X-ray crystallography, however the “resolution revolution” in cryo-electron microscopy^5,6^ will lead to a rapidly growing share of IMP structures determined by single-particle cryo-EM in the future (so far 5% of the PDB entries for all deposited “not unique” membrane proteins are already determined by EM). Soon after the mesophase crystallization technique has been introduced, lipidic cubic phase (LCP) crystallization has been positioned amongst the leading methods to be chosen for membrane protein crystallography^7^, complementing the use of bicelles^8^ and traditional vapour diffusion of protein-detergent complexes^3^. Furthermore, different lipoprotein nanoparticle systems have been developed to allow the reconstitution of membrane proteins into a lipid mimic environment suitable for EM, NMR, SAXS and functional studies. IMPs can be stabilized by a scaffold of saposin A proteins^9,10^ or inserted into nanodiscs composed of MSP1 scaffold proteins^11–14^ with positive consequences for its structural and functional characterization. However, for most methods investigating the structure of an IMP, the need to extract and solubilize IMPs from the membrane using detergents initially remains. Despite the increasing number of commercially available detergents, membrane protein crystal structures in the PDB are represented by a subset of detergents^15^. This might be due to biased selection of the detergents based on previous successes in the extraction of other membrane proteins or due to historical protocols in experienced membrane protein labs. The most commonly used detergent families in membrane protein crystallography are maltosides and glucosides, followed by amine oxides and polyoxyethylene glycols^3^. Altogether, crystallographic data support the use of n-Dodecyl β-D-maltoside (DDM), n-Decyl-β-D-Maltopyranoside (DM), Octyl-beta-glucoside (OG) and Lauryldimethylamine-N-oxide (LDAO). Besides crystallography, CYMAL and Fos-choline detergents have been shown to be very efficient in extracting inner membrane proteins, DM for outer membrane proteins and LDAO used for solubilization of transport proteins^15^. In summary, as a rule of thumb, detergents with longer acyl chains seem to be more efficient in solubilization and stabilization but shorter chain detergents form smaller micelles sizes, leading to a tighter packing in the crystal lattice and better diffraction^16,17^. The question would be, beside the purpose of crystallographic studies, are we on the right path by repeatedly employing these detergents? Thermal denaturation assays might help us to evaluate the most commonly used detergents over the last decade in membrane protein research.

While the link between protein thermodynamic stability and crystallization success for soluble globular proteins is not indeed absolute (many very stable proteins will never crystallize and some more unstable ones form crystals overnight), mono-dispersity and stability of a membrane protein has been postulated as a critical point for crystallization^18^. Instability of integral membrane proteins is indeed the bottleneck for structural and functional studies. α-helical membrane proteins could be partially unfolded when solubilized with detergents, maintaining the helical structure of the transmembrane domains but losing the tertiary inter-helix interactions^19^. In addition, the removal of specific lipids during solubilization and SEC, a process called delipidation, can severely affect protein stability and function^20–22^. Therefore, the initial condition used to extract an IMP from the membrane will mostly determine its half-life and stability throughout the entire downstream process.

Several methods exist for assessment of protein stability of IMPs. GFP-tagged proteins can be monitored by fluorescent-detection SEC to address their aggregation state during expression and purification^23,24^. In addition, stability in detergents has been studied using analytical size exclusion chromatography^18,25^. Slotboom *et al*. have addressed the behavior of IMPs in detergent micelles and lipid/detergent micelles in SEC-LS (light-scattering) analysis compared to sedimentation equilibrium centrifugation experiments^26^. Other methods like differential filtration and ultracentrifugation assays^4^ address the stability by quantifying the remaining protein in solution. However, the lack of aggregation is not necessarily an indication for a properly folded protein. The thermal denaturation assay using the thiol-specific fluorochrome N-[4-(7-diethylamino-4-methyl-3-coumarinyl) phenyl-maleimide (CPM)^27^ links stability of the IMP to the accessibility of free cysteine residues that become exposed to the solvent upon denaturation. However, not all membrane proteins are suitable for this analysis due to the lack of compatible Cys residues. Thermal denaturation experiments provide information whether an IMP displays a cooperative unfolding transition following a two-state model^28^, providing evidence of the folded state. The inflection point in a standard denaturation curve, T_m_, has been a recurrent parameter to compare protein thermodynamic stability. Other studies have evaluated the stability of membrane proteins following the change of intrinsic fluorescence during thermal denaturation using a differential scanning fluorimetry device for the screening of lipid-like peptides^29^ and ligands^30–33^. Recently, Nji *et al*. have developed an engineered thermal-shift screen for detecting IMP lipids preferences based on fluorescence-detection size-exclusion chromatography (FSEC-TS). By using GFP-fusion IMPs the authors compare the stabilization of bacterial and eukaryotic membrane proteins before and after purification, and after addition of selected lipids to the purified IMPs. The authors conclude that eukaryotic membrane proteins appear to be more sensitive to the presence of lipids than prokaryotic ones^21^.

In this work, we use the nanoDSF technology (DSF) to study the unfolding of membrane proteins, following the intrinsic fluorescence of tryptophan residues during a thermal ramp in a detergent screen with 94 different detergents. In addition to the melting temperature, this device allows to follow the onset of aggregation by monitoring static light scattering. We describe a fast, high-throughput and quantitative methodology for screening detergents based on dilution rather than buffer exchange. The samples are diluted from its original extraction condition to 94 commercially available detergents with no exchange needed to observe a stabilization or destabilization effect. This will guide the selection of the appropriate detergent for further downstream processing of the IMP.

## Results

### Stability of selected integral membrane proteins in DDM

We have selected nine membrane proteins belonging to different families and originating from different species (see Table 1). Four IMPs belong to the major facilitator superfamily of transporters; DtpA, DgoT, and MdfA involved in nutrient and drug transport^33–38^, and LacY is the well-characterised lactose permease^39^. Ij1 is an ABC-transporter involved in ion transport. Kv1 is an IMP of unknown function and structure from the pathogenic bacterium *Pseudomonas aeruginosa* and Im1 is a prokaryotic kinase with two transmembrane domains. As an eukaryotic example, we have included the human P2X4 receptor from the P2X ionotropic receptors family, a regulator in neuropathic pain^40^. In addition, we screened bacteriorhodopsin (BR) from *Halobacterium salinarum* as a control example of a well-characterized membrane protein^41^.

**Table 1:** IMPs used for the stability assay.

Protein	Organism	Family	Function	Number of Trp residues	PDB ID
DgoT	*E*. *coli*	MFS transporters	putative galactonate transporter	14	6E9N, 6E9O
MdfA	*E*. *coli*	MFS transporter	multi drug resistance	9	4ZP0, 4ZOW, 4ZP2, 6GV1, 6EUQ
DtpA	*E*. *coli*	MFS transporter	peptide transporter	10	6GS1, 6GS4, 6GS7
Kv1	*Pseudomonas aeruginosa*	unknown	unknown	17	—
Ij1	*E*. *coli*	ABC-Transporter	ion transport	22	—
P2X4	*Homo sapiens*	P2X ionotropic receptors	regulator in mediating neuropathic pain	6	4DW0, 4DW1 (zebrafish)
BR	*Halobacterium salinarum*	7TM receptor	proton pump	8	4MD1, 4MD2, 4XXJ
LacY	*E*. *coli*	MFS transporter	transport of beta-galactosides	5	1PV6
Im1	*E*. *coli*	HisKA	Kinase	2	—

**Figure 1:**
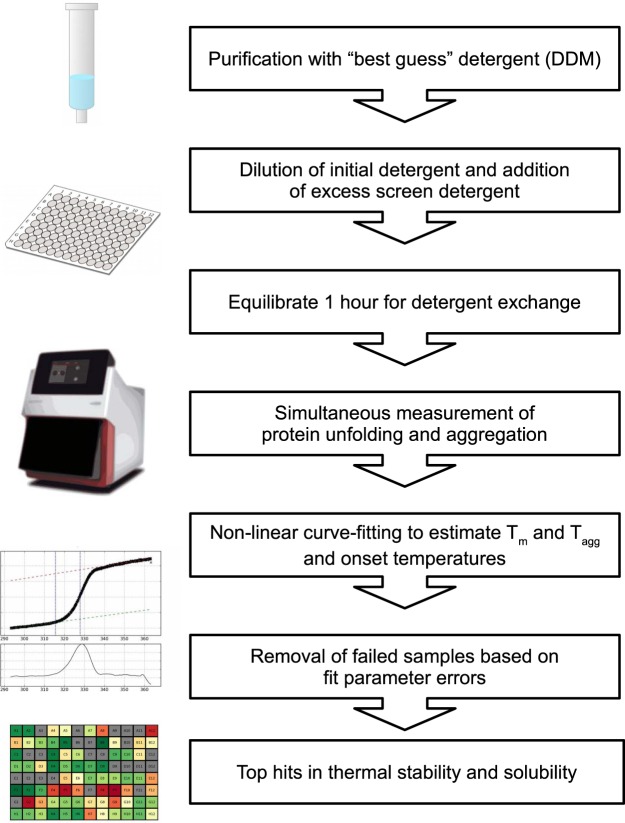
Flow chart of the thermostability assay. Solubilized and purified proteins in DDM (see Supplementary Table 1 for DDM concentrations for each protein) are diluted tenfold to a 94 detergent screen followed by DSF and scattering measurements. Apparent melting and mid-aggregation temperatures (T_m_ and T_agg_ respectively) are estimated after curve fitting. Successful measurements for the thermodynamic parameters are plotted using a color code (red “denatured” and green “native”) for visual identification of stabilization conditions.

We have solubilized the isolated membranes in 1–2% DDM, usually the first choice detergent of most labs working with IMPs due to its rather mild denaturation properties. All proteins were expressed and purified as described in Material and Methods using DDM as starting detergent, with size exclusion chromatography (SEC) as last purification step, concentrated using centrifugal filter devices (Supplementary Fig. 1), and then diluted tenfold to the detergent screen covering various detergent families used for membrane solubilization and purification (Fig. 1 and Table S1). Importantly, for the proteins Kv1 and P2X4 concentrators with a cutoff smaller than the DDM micelle size (59.5 KDa) have been used, leading to samples with increased detergent concentration. In the case of BR, 1% OG was used as the starting condition as an external control due to its compatibility with crystallization^42,43^.

### Detergent screening

**Figure 2:**
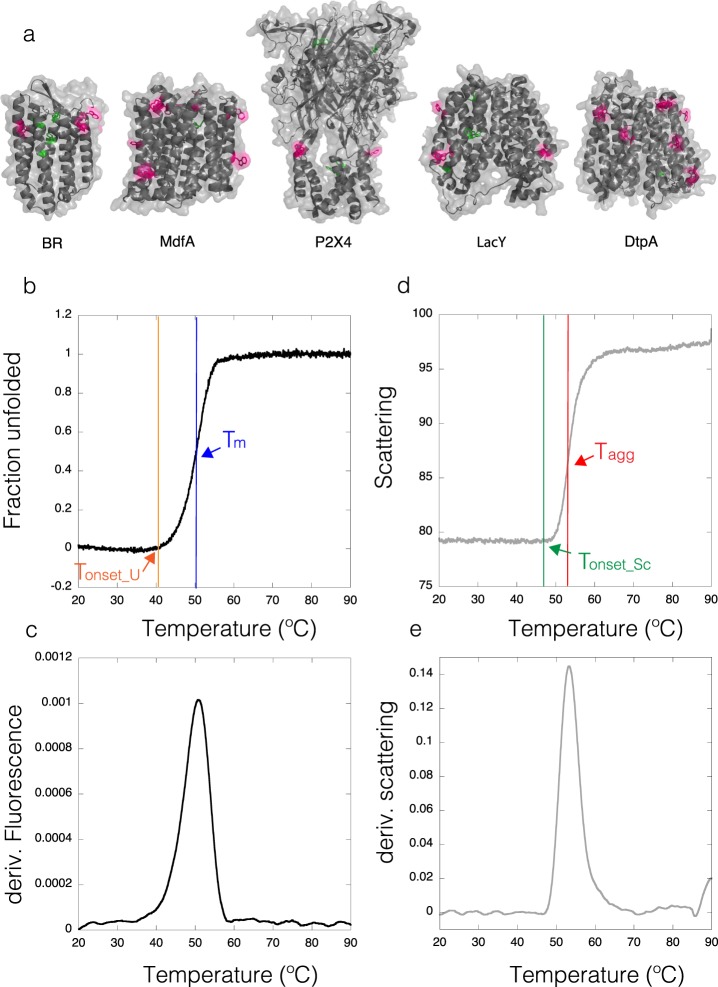
Thermal denaturation of IMPs. (**a**) Cartoon representations for *Halobacterium salinarum* Bacteriorhodopsin (BR) PDB:4XXJ, peptide transporter DtpA (DtpA) PDB: 6GS1, multidrug transporter MdfA (MdfA) PDB:4ZP0, human P2X4 ion channel (P2X4) based on PDB:4DW1 and lactose permease (LacY) PDB:1PV6. Surface exposed Trp residues are highlighted in pink while buried ones are highlighted in green. (**b**) Example of a thermal denaturation curve (baseline corrected) and the visualization of T_m_ at the inflection point and the change in the slope reporting T_onset_U_. (**c**) First derivative of the DSF curve with the maximum reporting T_m_. (**d**) Scattering curve and the visualization of T_agg_ and T_onset_Sc_. (**e**) First derivative from the scattering curve with the maximum reporting T_agg_ (comparable to 50% of the molecules in an aggregated state).

**Figure 3:**
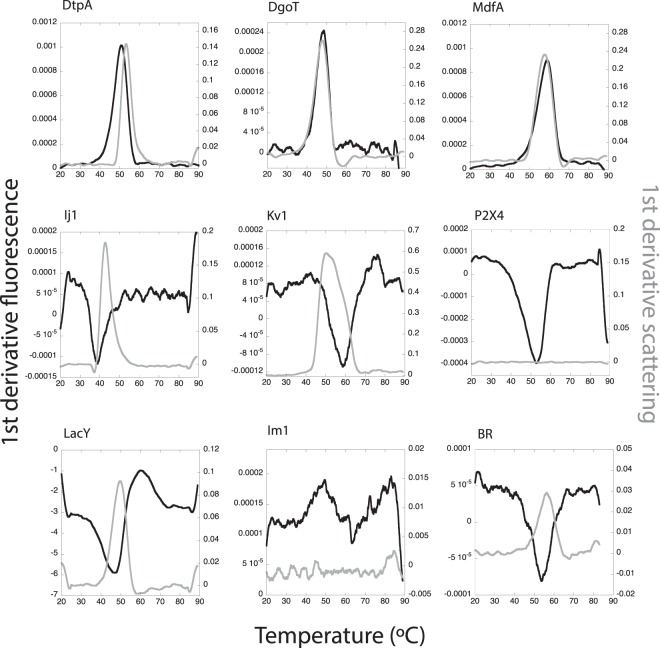
Stability of all IMPs at the starting condition. First derivatives of the DSF (black) and scattering (grey) curves are shown as a function of temperature for condition A2. All proteins display clear DSF unfolding transitions with exception of Im1. Scattering data for P2X4 and Im1 did not display transitions.

In order to test the thermodynamic stability of IMPs after being isolated from their natural environment and solubilized in detergents, we have performed a high-throughput analysis by DSF and light scattering. In IMPs, tryptophan residues (Trp) are either buried in the hydrophobic core of the protein, or exposed to the micelle belt after solubilization with detergents (Fig. 2a and Supplementary Fig. 2a). Thus, upon denaturation, IMPs could display shifts of the center of mass of the Trp fluorescence spectrum in both directions (blue and red)^44^. Fluorescence transition curves measured at 330 nm and 350 nm (and the ratio 350/330) result in “S curves” with positive slopes, or “Z curves” with negative slopes (Supplementary Fig. 3), from which the thermodynamic parameters like the melting temperature (T_m_) and the onset of unfolding (T_onset_U_) can be extracted (Fig. 2b,c; and Supplementary Fig. 2b). For the high-throughput analysis we used the fluorescence ratio (350/330) to obtain T_m_ (see Material and Methods) with the exception of LacY and BR where the ratio was less informative than the fluorescence measured at 330 nm (Supplementary Fig. 3). Figure 3 shows the first derivatives from the DSF curves obtained for the thermal denaturation at the starting condition, prior to dilution into the detergent screen. The selected nine IMPs display different thermal stabilities (represented by different T_m_) at the starting point of the assay with a range between 38.9 °C and 59.3 °C (Table 2). Most proteins show a clear transition between the native and the unfolded state (Supplementary Fig. 3 and Fig. 3, black curves). In the case of LacY, the Ratio curve contained no unfolding transtions as judged by the absence of a peak in the smoothened first derivative curve. At the same time F330 and F350 of LacY showed a clear transition with a similar shape, so we used F330 due to higher signal strength (Supplementary Fig. 3). Finally, Im1 seems either to be unfolded after purification or its two Trp residues are not suited to report the unfolding of the protein.

**Table 2:** Melting and aggregation temperatures for the standard condition (A2) before dilution to the detergent screen.

Protein	T_m_	T_agg_	T_onset_U_	T_onset_Sc_	n DSF	n Sc
DtpA	50.1 ± 0.2	52.9 ± 0.9	40.9 ± 0.2	46.0 ± 1.4	2	2
DgoT	48.1 ± 0.01	47.6 ± 0.1	40.0 ± 0.1	38.7 ± 0.1	2	2
LacY	46.2 ± 0.1	49.6 ± 0.1	37.0 ± 0.3	41.0 ± 0.1	1	2
Kv1	59.3 ± 0.2	53.5 ± 0.2	47.6 ± 0.5	37.7 ± 0.3	2	2
Ij1	38.9 ± 0.2	43.9 ± 0.2	27.2 ± 0.5	35.9 ± 0.2	2	2
MdfA	58.5 ± 0.1	57.9 ± 0.1	46.6 ± 0.3	48.0 ± 0.1	2	2
P2X4	51.5 ± 1.4	NA	36.8 ± 1.0	NA	4	0
BR	54.9 ± 0.1	57.2 ± 0.1	40.3 ± 0.1	46.0 ± 0.1	4	4

Where T_m_ is the melting temperature and T_agg_ the mid-aggregation point obtained by curve fitting from the DSF and scattering curves respectively. T_onset_U_ relates to the change in the slope, equivalent to the temperature where 1% of protein becomes unfolded (U) or aggregated (Sc). n indicates the number of replicates. Average and standard deviation were computed from the replicates.

In parallel to the DSF measurements, static light scattering has been recorded in our Prometheus device equipped with a backscattering module^45^ which enables monitoring the onset of aggregation (T_onset_Sc_) of the protein and the calculation of an aggregation mid-point (T_agg_) (Figs 2d,e and 3, grey curves). Aggregation transitions were not detected for two proteins from the dataset (P2X4 and Im1). The absence of a scattering transition for Im1 would agree with either the hypothesis of an unfolded protein, or due to the protein concentration used because of limited material available. For the P2X4 receptor, experiments were performed at 0.15 and 0.25 mg/ml and no scattering transition could be detected for unknown reasons.

In some cases, as for DgoT, both the unfolding and scattering transitions, T_m,_ and T_agg_, are similar (Fig. 3) reporting coupled unfolding and aggregation. In other cases, however, e.g. for Ij1, the T_m_ is lower than T_agg_ indicating that the protein starts to unfold, leading to macroscopic aggregation.

We next analyzed the behavior of the different IMPs after tenfold dilution to other detergents to see whether we are able to monitor the stabilizing and destabilizing properties of particular detergents by dilution instead of purifying the protein in individual detergents from the beginning. We have incubated the IMPs in the screening conditions for one hour. Our control experiments for addressing the equilibration of the detergent mix indicate that this time is sufficient to obtain consistent and reproducible results (Supplementary Fig. 4). To address how dilution into a new detergent condition compares to methods for detergent exchange, we have performed experiments after chromatographic detergent exchange and compared the inflection points from these DSF curves with those obtained after the 10-fold dilution (Supplementary Fig. 5). Our experiments show that dilution is a good indicator for whether the IMP would be stabilized or destabilized in the presence of the “new detergent”.

**Figure 4:**
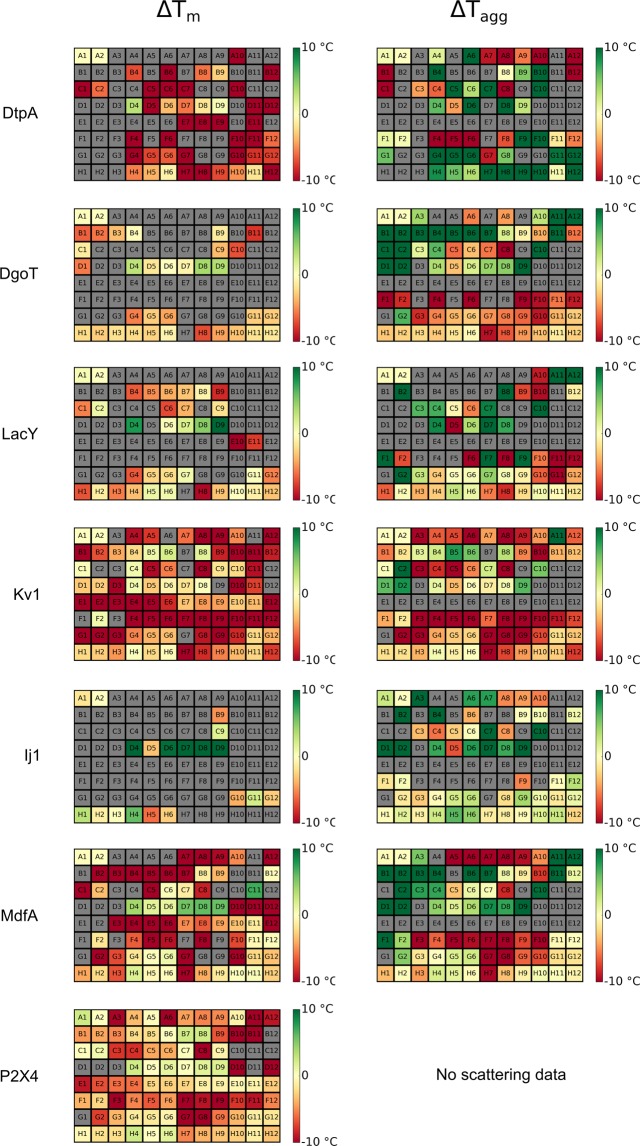
Heat-maps for delta T_m_ and T_agg_. Heat-maps for delta T_m_ and T_agg_. Red/yellow/green wells are colored according to the difference between the sample and original detergent (screen position A2) with difference 10 °C and above being green and −10 °C and below being red. Yellow corresponds to 0 °C (no difference compared to the the original detergent). Grey wells indicate samples that could not be analyzed.

Figure 4 represents heat maps for both DSF and scattering data. The data are presented as changes in T_m_ and T_agg_ compared to the stability of the starting point (position A2). Our high-throughput stability analysis (initially) compares the observed T_m_ values in different detergents.

As a control experiment, we tested if the detergent screen *per-se* could introduce background fluorescence or scattering upon heating that could be misinterpreted as an unfolding or aggregation transition. Even though detergent fluorescence contributed up to 25% of the overall signal in some cases, none of the detergents showed a sigmoidal curve in the F350/F330 ratio in the absence of protein. However, strong scattering signal similar to aggregation transitions were detected for PEG derivatives, ethylene glycol alkyl ethers, Tween-20, NID-P40, Triton X100 and Triton X115 (Supplementary Table 2). Consequently, these conditions were removed from the subsequent analysis when it has not been possible to distinguish whether the protein or the detergent contributed to the scattering signal.

### Targets stabilized by long chain maltoside detergents

**Figure 5:**
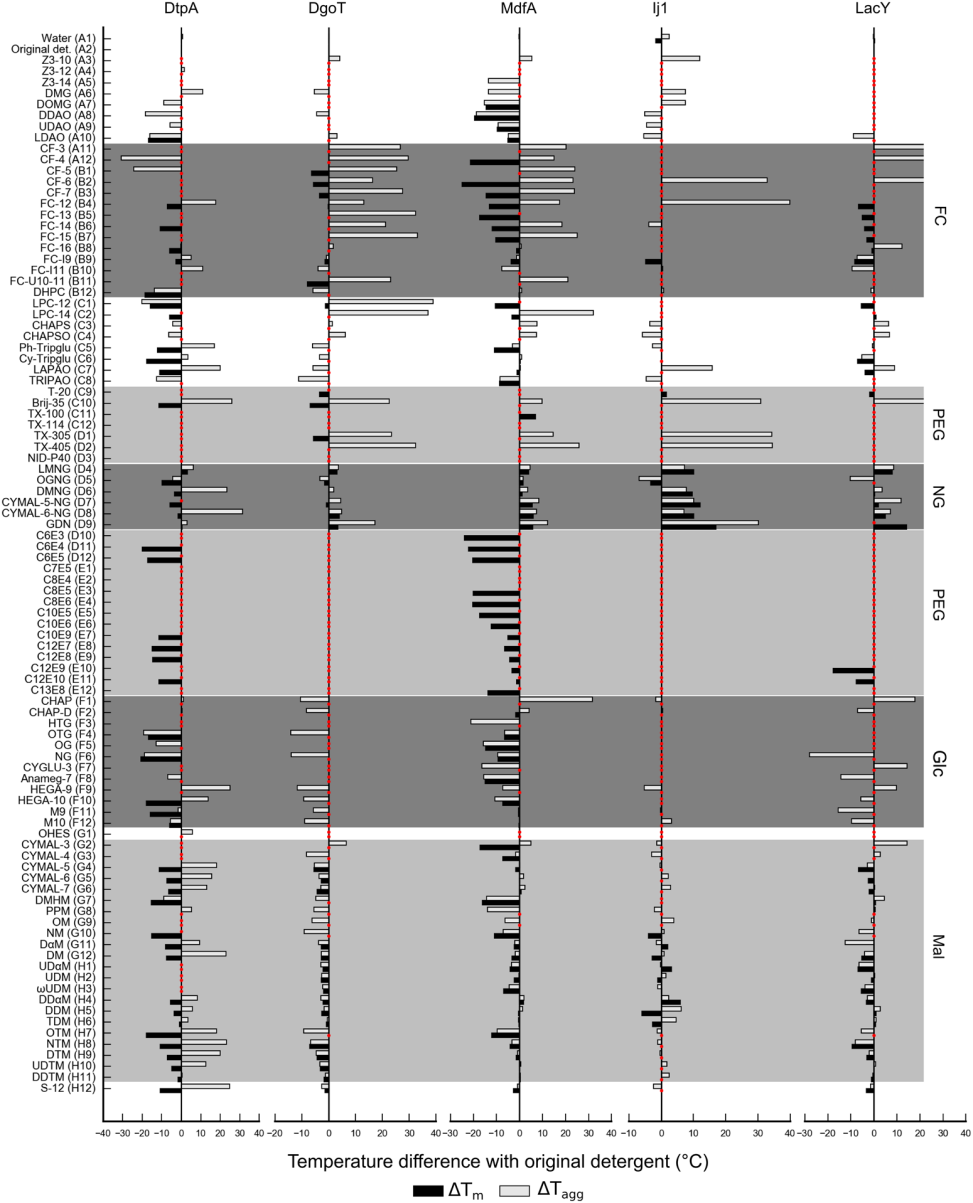
Bar graphs representing changes in T_m_ (black) and T_agg_ (grey) for transporters. T_m_ is calculated from the fluorescence ratio F350/F330 data with exception of LacY, where fluorescence at 330 nm was used. Red dots correspond to conditions that could not be fitted. Several prominent detergent families are highlighted: fos-cholines (FC), polyethyleneglycol (PEG), neopentyl-glycol (NG), glucose (Glc) and maltose (Mal) based detergents.

All transporter proteins analyzed in this study (DtpA, DgoT, MdfA, LacY and Ij1) are stabilized by detergents from the maltose-NG family (Figs 4 and 5). Specifically, the detergent Lauryl Maltose Neopentyl Glycol (LMNG) (Table 3), represented in position D4, generated positive shifts in both T_m_ and T_agg_ for all five transporters. In addition, detergent GDN-101 (position D9) has been the second best stabilizing detergent. DtpA has been the most destabilized protein after its dilution in the detergent screen, indicating that the IMP was already very stable in DDM. Consistently, OGNG (Glucose-NG; position D5) destabilized all analyzed transporters. For MdfA and Ij1, n-Dodecyl-α-D-Maltopyranoside (α-DDM) seems to be a better stabilizer than the mostly used and cheaper version DDM (n-Dodecyl-β-D-maltoside). Indeed, alpha isomers of the maltosides display positive hits (DαM, UDαM and DDαM) for Ij1 (Fig. 5). Finally, detergents belonging to the PEG family have shown a major destabilizing effect resulting in lower T_m_ or curves without transitions indicating that the proteins are likely denatured at room temperature in this detergent class (Fig. 5).

**Table 3:** Detergent screen.

Screen		Chemistry	Detergent	Abbreviation	CMC (mM)	Conc (mM)	Micelle size (kDa)
A	1	—	Water				
A	2	—	Blank (for solubilizing detergent)				
A	3	Propanesulfonate	Anzergent 3-10	Z3-10	39	78	13
A	4	Propanesulfonate	Anzergent 3-12	Z3-12	2.8	8.4	23.5
A	5	Propanesulfonate	Anzergent 3-14	Z3-14	0.2	10	39
A	6	Dimethylglycine	n-Decyl-N,N-Dimethylglycine	DMG	19	38	
A	7	Dimethylglycine	n-Dodecyl-N,N-Dimethylglycine	DOMG	1.5	4.5	
A	8	Dimethylamine-oxide	n-Decyl-N,N-Dimethylamine-N-Oxide	DDAO	10.5	21	1.4098
A	9	Dimethylamine-oxide	n-Undecyl-n,n,-Dimethylamine-Oxide	UDAO	3.2	9.6	
A	10	Dimethylamine-oxide	n-Dodecyl-N,N-Dimethylamine-N-Oxide	LDAO	1	3	17.4
A	11	Phosphocholine	CyclofosTM-3	CF-3	43	86	
A	12	Phosphocholine	Cyclofos-4	CF-4	14	28	
B	1	Phosphocholine	Cyclofos-5	CF-5	4.5	13.5	
B	2	Phosphocholine	Cyclofos-6	CF-6	2.68	8.04	
B	3	Phosphocholine	Cyclofos-7	CF-7	0.62	6.2	
B	4	Phosphocholine	Fos-Choline-12	FC-12	1.5	4.5	19
B	5	Phosphocholine	Fos-Choline-13	FC-13	0.75	7.5	32
B	6	Phosphocholine	Fos-Choline-14	FC-14	0.12	6	44
B	7	Phosphocholine	Fos-Choline-15	FC-15	0.07	7	52
B	8	Phosphocholine	Fos-Choline-16	FC-16	0.013	1.3	73
B	9	Phosphocholine	Fos-Choline-ISO-9	FC-I9	32	64	
B	10	Phosphocholine	Fos-Choline-ISO-11	FC-I11	26.6	53.2	
B	11	Phosphocholine	Fos-Choline-UNSAT-11-10	FC-U10-11	6.2	15.5	
B	12	Phosphocholine	1,2-Diheptanoyl-sn-Glycero-3-Phosphocholine	DHPC	1.4	4.2	
C	1	Phosphocholine_lyso	LysoPC-12	LPC-12	0.7	7	
C	2	Phosphocholine_lyso	LysoPC-14	LPC-14	0.036	3.6	
C	3	Propanesulfonate	CHAPS	CHAPS	8	20	7
C	4	Propanesulfonate	CHAPSO	CHAPSO	8	20	7
C	5	Glycotripod	Ph-Tripglu	Ph-Tripglu	3.6	10.8	
C	6	Glycotripod	Cy-Tripglu	Cy-Tripglu	1.8	5.4	
C	7	Dimethylamine-oxide	LAPAO	LAPAO	1.6	4.8	37.8
C	8	Dimethylamine-oxide	Tripao	TRIPAO	4.5	13.5	
C	9	Polyoxyethylene	Anapoe-20 (Tween 20)	T-20	0.059	5.9	
C	10	Polyoxyethylene	Anapoe-35 (Brij-35)	Brij-35	0.091	9.1	47.92
C	11	Polyoxyethylene	Anapoe-X-100	TX-100	0.23	11.5	80
C	12	Polyoxyethylene	Anapoe-X-114	TX-114	0.2	10	
D	1	Polyoxyethylene	Anapoe-X-305	TX-305	0.65	6.5	
D	2	Polyoxyethylene	Anapoe-X-405	TX-405	0.81	8.1	
D	3	Polyoxyethylene	[Octylphenoxy]Polyethoxyethanol	NID-P40	0.3	15	60–90
D	4	Maltose_NG	Lauryl Maltose Neopentyl Glycol	LMNG	0.01	1	93
D	5	Glucose_NG	Octyl Glucose Neopentyl Glycol	OGNG	1.02	3.06	
D	6	Maltose_NG	Decyl Maltose Neopentyl Glycol	DMNG	0.036	3.6	
D	7	Maltose_NG	CYMAL-5 Neopentyl Glycol	CYMAL-5-NG	0.058	5.8	
D	8	Maltose_NG	CYMAL-6 Neopentyl Glycol	CYMAL-6-NG	0.02	2	
D	9	Maltose_NG	GDN-101	GDN	0.018	1.8	80
D	10	Polyoxyethylene	Triethylene Glycol Monohexyl Ether	C6E3	23	46	
D	11	Polyoxyethylene	Tetraethylene Glycol Monohexyl Ether	C6E4	30	60	
D	12	Polyoxyethylene	Pentaethylene Glycol Monohexyl Ether	C6E5	37		
E	1	Polyoxyethylene	Pentaethylene Glycol Monoheptyl Ether	C7E5	21	42	
E	2	Polyoxyethylene	Tetraethylene Glycol Monooctyl Ether	C8E4	8	20	25
E	3	Polyoxyethylene	Pentaethylene Glycol Monooctyl Ether	C8E5	7.1	17.75	
E	4	Polyoxyethylene	Hexaethylene Glycol Monooctyl Ether	C8E6	10	25	13
E	5	Polyoxyethylene	Pentaethylene Glycol Monodecyl Ether	C10E5	0.81	8.1	28
E	6	Polyoxyethylene	Hexaethylene Glycol Monodecyl Ether	C10E6	0.9	9	
E	7	Polyoxyethylene	Polyoxyethylene(9)decyl Ether	C10E9	1.3	3.9	
E	8	Polyoxyethylene	Heptaethylene Glycol Monododecyl Ether	C12E7	0.069	6.9	
E	9	Polyoxyethylene	Octaethylene Glycol Monododecyl Ether	C12E8	0.09	9	67
E	10	Polyoxyethylene	Polyoxyethylene(9)dodecyl Ether	C12E9	0.05	5	83
E	11	Polyoxyethylene	Polyoxyethylene(10)dodecyl Ether	C12E10	0.1	10	
E	12	Polyoxyethylene	Polyoxyethylene(8)tridecyl Ether	C13E8	0.1	10	
F	1	Gluconamidopropyl	Big CHAP	CHAP	2.9	8.7	9
F	2	Gluconamidopropyl	Big CHAP, Deoxy	CHAP-D	1.4	4.2	13.5
F	3	Thioglucopyranoside	n-Heptyl-b-D-Thioglucopyranoside	HTG	29	58	8
F	4	Thioglucopyranoside	n-Octyl-b-D-Thioglucopyranoside	OTG	9	12.5	
F	5	Glucopyranoside	n-Octyl-b-D-Glucopyranoside	OG	18	36	19
F	6	Glucopyranoside	n-Nonyl-b-D-Glucopyranoside	NG	6.5	16.25	41
F	7	Glucopyranoside	CYGLU-3	CYGLU-3	28	56	
F	8	Glucopyranoside	HECAMEG	Anameg-7	19.5	39	30.9
F	9	Glucamide	Hega-9	HEGA-9	39	78	1.8275
F	10	Glucamide	Hega-10	HEGA-10	7	17.5	
F	11	Methylglucamide	Mega-9	M9	25	50	
F	12	Methylglucamide	Mega-10	M10	6	6	
G	1	Hydroxyethylsulfoxide	2-Hydroxyethyloctylsulfoxide	OHES	24.3	48.4	
G	2	Maltoside	CYMAL-3	CYMAL-3	30	60	3
G	3	Maltoside	CYMAL-4	CYMAL-4	7.6	19	12
G	4	Maltoside	CYMAL-5	CYMAL-5	2.4	7.2	23
G	5	Maltoside	CYMAL-6	CYMAL-6	0.56	5.6	53
G	6	Maltoside	CYMAL-7	CYMAL-7	0.19	9.5	80
G	7	Maltoside	2,6-Dimethyl-4-Heptyl-b-D-Maltoside	DMHM	27.5	55	
G	8	Maltoside	2-Propyl-1-Pentyl-b-D-Maltopyranoside	PPM	42.5	85	
G	9	Maltoside	n-Octyl-b-D-Maltopyranoside	OM	19.5	39	22
G	10	Maltoside	n-Nonyl-b-D-Maltopyranoside	NM	6	15	26
G	11	Maltoside	n-Decyl-a-D-Maltopyranoside	DαM	1.6	4.8	
G	12	Maltoside	n-Decyl-b-D-Maltopyranoside	DM	1.8	5.4	33
H	1	Maltoside	n-Undecyl-a-D-Maltopyranoside	UdαM	0.58	5.8	
H	2	Maltoside	n-Undecyl-b-D-Maltopyranoside	UDM	0.59	5.9	36
H	3	Maltoside	ω-Undecylenyl-b-D-Maltopyranoside	ωUDM	1.2	3.6	
H	4	Maltoside	n-Dodecyl-a-D-Maltopyranoside	DdαM	0.15	7.5	50
H	5	Maltoside	n-Dodecyl-b-D-Maltopyranoside	DDM	0.17	8.5	59.5
H	6	Maltoside	n-Tridecyl-b-D-Maltopyranoside	TDM	0.03	1.5	100
H	7	Thiomaltoside	n-Octyl-b-D-Thiomaltopyranoside	OTM	8.5	21.25	
H	8	Thiomaltoside	n-Nonyl-b-D-Thiomaltopyranoside	NTM	3.2	9.6	
H	9	Thiomaltoside	n-Decyl-b-D-Thiomaltopyranoside	DTM	0.9	9	37
H	10	Thiomaltoside	n-Undecyl-b-D-Thiomaltopyranoside	UDTM	0.21	10.5	55
H	11	Thiomaltoside	n-Dodecyl-b-D-Thiomaltopyranoside	DDTM	0.05	5	66
H	12	Sucrose	Sucrose-12	S-12	0.3	15	

CMC: critical micelle concentration (mM). Conc: final concentration in the assay (mM).

**Figure 6:**
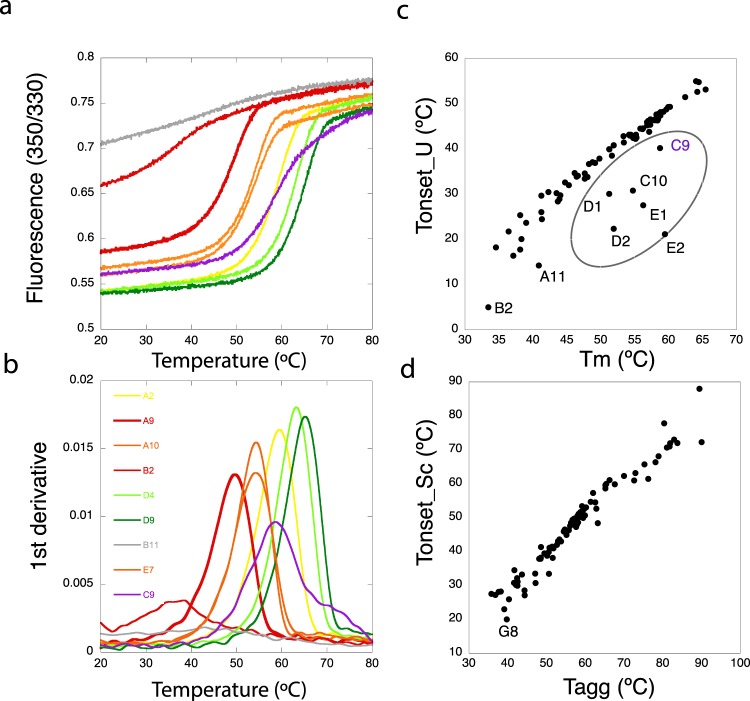
Comparison of T_m_ and T_onset_U_ as stability reporters. MdfA DSF transitions, fluorescence ratio (**a**) and first derivatives (**b**), in selected detergents: GDN-101 (green), LMNG (light green), 0.03% DDM (yellow, initial condition), LDAO and APO109 (orange), UDAO and cyclofos-6 (red), fos-choline U10-11 (grey) and Tween 20 (violet). (**c**) Scatter plot of T_m_ vs T_onset_U_. The circle highlights samples with atypically low T_onset_U_. (**d**) Scatter plot for T_agg_ vs T_onset_Sc_.

Figure 6a,b show selected DSF curves (raw data) and first derivatives for MdfA in selected detergents showing different stabilization/destabilization examples. Besides general effects reported by T_m_ (Figs 4 and 5), this study also enabled us to detect a linear relationship between inflection points (T_m_) and onset of unfolding temperatures (calculated from slopes). We have observed cases of DSF curves that exhibit a relative high T_m_ combined with low T_onset_U_ (Fig. 6). Samples displaying such behavior can be detected by a scatter plot of T_onset_U_ as a function of T_m_. In most cases these values are linearly correlated, while samples with low T_onset_U_ can be identified as outliers in this analysis (Fig. 6c). In such cases T_m_ would not be the best reporter for overall protein stability and T_onset_U_ should be taken into account. Interestingly, when analyzing the scattering signal, the linear relationship between T_agg_ and T_onset_Sc_ contained no outliers (Fig. 6d).

### Evaluation of fos-choline and and PEG family detergents for IMP solubilization

**Figure 7:**
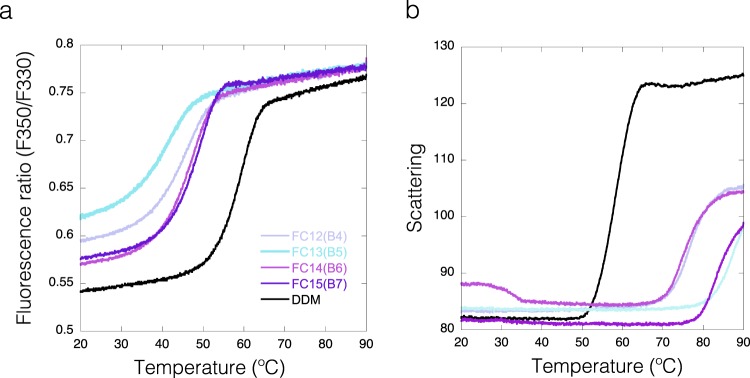
Fos-Choline enhances solubility of an unfolded MdfA. (**a**) DSF curves for MdfA in DDM (black) and in different Fos-choline detergents (Fos-Choline 12, 13, 14, 15 and 16; in the violet-cyan range). (**b**) Scattering curves, same colour code as for A.

Currently it is a debate in the field whether fos-choline detergents, known for being very efficient in solubilization, have denaturing effects on membrane proteins^46,47^. Indeed, based on our scattering data, detergents from the fos-choline family have a general positive effect in preventing aggregation (see T_agg_ heatmaps in Fig. 4 and grey bars in Fig. 5). But despite its improved solubility the fluorescence data on IMPs in fos-choline detergents give a different picture on the folding status of the protein. The derived T_m_ of unfolding is much lower than T_agg_ or in many cases an unfolding transition cannot be monitored (Fig. 5 black bars, Fig. 7). This indicates that IMPs in fos-choline detergents are strongly destabilized and only represent partially folded or unfolded states. This observation is demonstrated in Fig. 7, showing that MdfA starts to aggregate in fos-choline detergents at temperatures above 70 °C while the fluorescence data show the protein is already unfolded. In summary, fos-choline detergents can efficiently solubilize unfolded IMPs. A clear example would be observing conditions B5 to B8 for DgoT on the DSF and Scattering heatmaps (Fig. 4). After dilution to Fos-Choline 13, 14, 15 and 16 detergents; DgoT did not present DSF transitions (grey wells) while the scattering profiles for these conditions showed increased solubility with late aggregation profiles (green wells).

In addition, our assay shows that dilution to PEG family detergents retrieved destabilized or unfolded proteins displaying no DSF or Scattering transitions (Fig. 5). Remarkably, some members of this family (conditions C10, D1 and D2) enhanced the solubility of the IMPs while highly destabilizing them similarly to what was previously described for the fos-choline family (Figs 4 and 5).

### Correlation between IMP stability and micelle size

It has been previously addressed that smaller detergent micelle sizes are better suited for crystallographic studies of IMPs^23,48^, but at the same time the change to detergents with shorter acyl chains destabilizes IMPs and often leads to precipitation. Therefore, it is crucial to balance the length of the acyl chain of the used detergent with the stability of membrane proteins. We have calculated Spearman correlation coefficients to test if there is a monotonic relationship between the melting or aggregation temperatures and the size of the micelle (Table 4 and Supplementary Fig. 6). This coefficient does not make any assumptions about the underlying dependence between the variables, but shows how consistently one variable increases as the other one increases or decreases^49^. The number of data points used to calculate these correlation coefficients depends on the available information on micelle size in kDa for that particular detergent (Table 3) and reliable unfolding data for a given IMP in that detergent. For 5 out of 9 proteins the coefficient was >0.7, so the reported T_m_ is predominantly increasing with the micelle size. For 3 proteins the coefficient was around 0.4, which implies a weaker though still valid correlation. For 1 protein (due to incomplete dataset) no correlation was found. Overall, our results indicate that there is a positive correlation between the size of the detergent micelle and the thermodynamic stability of the IMP.

**Table 4:** Correlation between micellar size and T_m_ or T_agg_.

Sample	Readout	n	Spearman’s ρ
DtpA	Ratio	21	0.70
	Scattering	27	0.24
DgoT	Ratio	15	0.43
	Scattering	33	0.25
LacY	F330	8	0.00
	Scattering	27	-0.04
Kv1	Ratio	38	0.46
	Scattering	37	0.66
Ij1	Ratio	8	0.45
	Scattering	29	0.65
Im1	Ratio	9	0.78
MdfA	Ratio	33	0.74
	Scattering	36	0.20
P2X4	Ratio	42	0.80
BR	F330	28	0.84
	Scattering	28	0.73

Where n in the number of data points used to calculate the correlation coefficient. Coefficients approaching zero show no correlation between variables while those approaching 1 indicate a positive correlation (Y values increase as the X values increase).

### Implications for sample optimization

**Figure 8:**
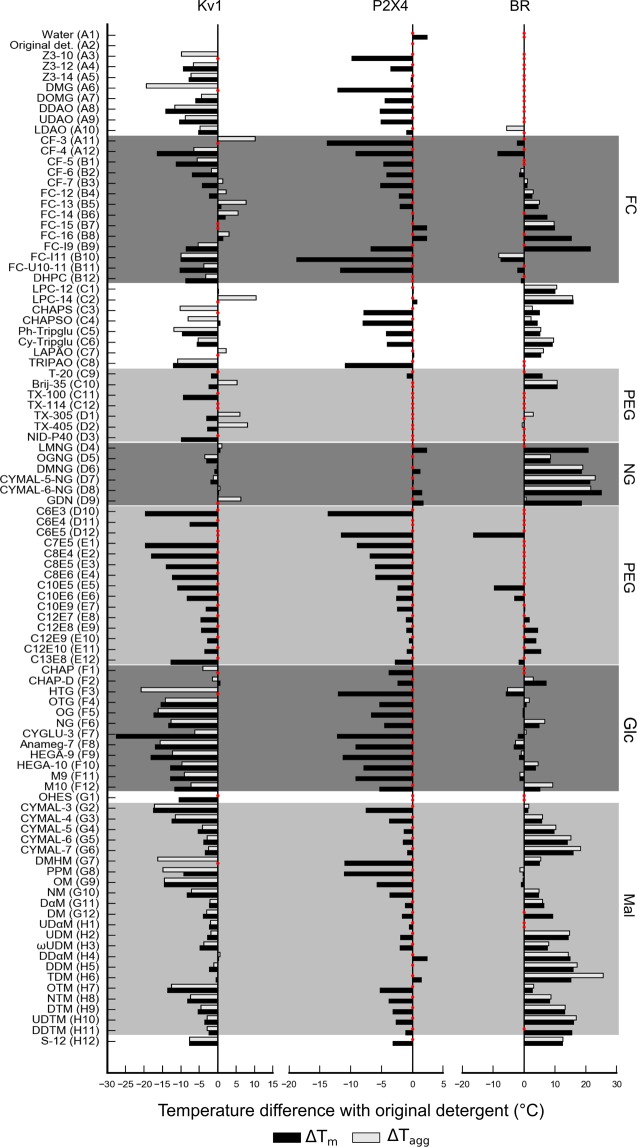
Bar graphs representing changes in T_m_ (black) and T_agg_ (grey) for Kv1, P2X4 and BR.T_m_ is calculated from the fluorescence ratio F350/F330 data with exception of BR, where fluorescence at 330 nm was used. Red dots correspond to conditions that could not be fitted properly. Several prominent detergent families are highlighted: fos-cholines (FC), polyethyleneglycol (PEG), neopentyl-glycol (NG), glucose (Glc) and maltose (Mal) based detergents. Note that the concentration used of P2X4 was not high enough to record scattering data.

Here we compare cases of IMPs that show improved, intermediate or poor general stabilization upon dilution in the detergent screen. How the protein will behave in the assay mainly depends on the stability in the starting condition. Kv1 is already stable in DDM and none of the screened detergents stabilizes the protein further (Fig. 8). Furthermore, the plot T_m_ vs T_onset_U_ does not show any outliers (Supplementary Fig. 7a). P2X4 shows positive hits for Maltose-NG detergents, DDαM and TDM, suggesting that an exchange of detergent for further downstream processing could be beneficial. BR (only IMP originally kept in OG) presents the largest positive changes in T_m_ after dilution to the detergent screen (Fig. 8). It is known that BR is less stable in OG compared to DDM, however OG is a good choice for crystallization due to its small micelle size. Once the starting condition of BR is changed to DDM, the result of the detergent screening approach changes, displaying a general destabilization effect (Supplementary Fig. 7b).

An interesting observation is the case of Im1 that does not have a transition of unfolding detectable by DSF (Fig. 3), most likely because the protein is misfolded. However, our assay shows some conditions displaying DSF transitions after detergent dilution (see Supplementary Fig. 8). These results suggest that a detergent substitution in an early stage of the purification could be beneficial. However, the sample so far has not been suitable for performing structural studies and different strategies for protein expression and solubilization need to be considered.

## Discussion

In this study we have measured the thermal stability of nine IMPs against a panel of 94 detergents by monitoring the intrinsic fluorescence of each IMP and its scattering properties. We conclude that the T_m_ calculated from the DSF measurements is a more stringent selection criterion than T_agg_ from the recorded scattering curves. We observe that in a number of cases, especially for PEG and fos-choline detergents, stabilization hits for T_agg_ are not accompanied by a higher T_m_. As a general observation, to obtain reliable scattering data higher protein concentrations (>0.15 mg/ml) are required, while for most of the IMPs tested, the fluorescence ratio obtained at 0.1 mg/ml produced reliable and reproducible unfolding data. In addition, whenever the ratio (F350/F330) cannot be used for stability determination, the fluorescence at 330 nm or 350 nm could be used to extract the thermodynamic parameters. Trp residues are often present in transmembrane domains of IMPs, and are excellent fluorophores in reporting changes in the environment upon changes in the folding state. Therefore, no additional dyes as used in other applications are necessary. In addition, analyzing T_m_ vs. T_onset_U_ could reveal conditions with similar melting temperatures but an earlier onset of unfolding indicates reduced stability.

During sample preparation, both T_m_ and T_agg_ should be taken into account and proteins should be kept below the lowest temperature at which anything deleterious happens.

Our workflow starts with a membrane protein that has been purified in DDM that is subsequently 10-fold diluted into the test detergent buffer. The fact that our workflow does not require additional chromatographic or filtration detergent exchange allows faster and less expensive screening. However, the dilution step brings residual DDM (bound to the IMP) that remains in the assay. Bound residual DDM could have different stabilizing or destabilizing effects on the IMP, which are translated into small perturbances in the observed T_m_. However, the scope of the assay is not to obtain the absolute melting temperatures in a given detergent but a reference system to compare detergents. Our results suggest that it is not necessary to go through a complete detergent exchange but a 10-fold dilution into a new detergent is sufficient to monitor the influence of other detergents on the protein stability. Our screening protocol allowed us to measure the stability and solubility of IMPs in a high-throughput manner and to determine the detergent or class of detergents that could be used (or should be avoided) when working with each system. Residual detergent concentration from the starting condition can affect the output of the assay. Therefore, we recommend researches to perform experiments considering the residual detergent form the dilution present in the assay to determine what would be the best working condition for each IMP. The ideal detergent would solubilize the IMP from the membrane in its native conformation and form a stable complex throughout purification. However, it has been discussed whether membrane protein stability is an intrinsic feature of the membrane protein and not detergent specific^23^. This would mean a stable IMP would be robust in more detergents compared to an unstable IMP. In our study, we have observed that when a membrane protein is quite stable in DDM it would be most likely destabilized by diluting it in many others. In contrast, a stable protein like BR could be further stabilized by other detergents when the starting condition has not been as favorable.

In our assay we detected detergents with general stabilizing effects. DDM, empirically the first chosen detergent in most labs working on IMPs, seemed to be a very good starting condition for extraction, solubilization and purification of folded membrane proteins. Also, LMNG seems to be a good stabilizer for all the cases tested in our study, and the maltose-NG family stabilized all transporters tested. For the detergent screen used, the diversity of the tested detergent chemistries showed that members of the PEG family do not improve the stabilization of our selected targets. We have now reported cases of detergents that contribute to the scattering signal and that fos-choline detergents keep unfolded IMPs in solution.

Our assay also quantified the correlation between thermal stability of an IMP in a detergent with its micelle size. Regarding crystallization, shorter chain detergents are preferred as they allow for better crystal packing and better diffracting crystals^50^. The goal is to find the shortest possible detergent that does not cause the protein to unfold^16,17^. Although shorter detergents would be more suitable for crystallography, a more stabilized protein in longer detergents could be beneficial for performing activity tests or reconstitution into scaffold systems such as nanodiscs or SapNPs. Lipids play a major role in preserving the native environment of membrane proteins. It has been shown for example that basal ATPase activity in ABC transporters purified in DDM micelles is lost but regained when reconstituted in liposomes or lipid bilayers^22^. It is of general knowledge that eukaryotic membrane proteins are on average less stable than their bacterial homologues after extraction with detergents. This is probably mainly due to delipidation. In our case, P2X4 is quite stable in DDM (T_m_ around 51 °C) however it could only be purified in the presence of 0.005% cholesteryl hemisuccinate (CHS). Therefore, we are working with a mix of detergent and CHS (see Material and Methods).

Finally, we propose that the described assay should be applied as an iterative process for membrane protein stabilization during purification. Once the IMP is solubilized in an appropriate detergent, addition of lipids could play a critical role in further stabilization. We propose to start with a first trial purification in DDM for a new IMP and then apply the described protocol with the most commonly used detergents (no need to screen all 94 detergents). The goal is to obtain a properly folded protein with sufficient stability to succeed in structural and functional studies.

## Methods

### Protein constructs

#### DgoT and DtpA

The DgoT and DtpA genes were cloned into the pNIC-CHTF vector (Addgene plasmid-ID Plasmid #26105)^51^ using LIC cloning^52,53^.

#### DgoT protein sequence including tags

MDIPVNAAKPGRRRYLTLVMIFITVVICYVDRANLAVASAHIQEEFGITKAEMGYVFSAFAWLYTLCQIPGGWFLDRVGSRVTYFIAIFGWSVATLFQGFATGLMSLIGLRAITGIFEAPAFPTNNRMVTSWFPEHERASAVGFYTSGQFVGLAFLTPLLIWIQEMLSWHWVFIVTGGIGIIWSLIWFKVYQPPRLTKGISKAELDYIRDGGGLVDGDAPVKKEARQPLTAKDWKLVFHRKLIGVYLGQFAVASTLWFFLTWFPNYLTQEKGITALKAGFMTTVPFLAAFVGVLLSGWVADLLVRKGFSLGFARKTPIICGLLISTCIMGANYTNDPMMIMCLMALAFFGNGFASITWSLVSSLAPMRLIGLTGGVFNFAGGLGGITVPLVVGYLAQGYGFAPALVYISAVALIGALSYILLVGDVKRVGAENLYFQ\SHHHHHHDYKDDDDK.

#### DtpA protein sequence including tags

MSTANQKPTESVSLNAFKQPKAFYLIFSIELWERFGYYGLQGIMAVYLVKQLGMSEADSITLFSSFSALVYGLVAIGGWLGDKVLGTKRVIMLGAIVLAIGYALVAWSGHDAGIVYMGMAAIAVGNGLFKANPSSLLSTCYEKNDPRLDGAFTMYYMSVNIGSFFSMIATPWLAAKYGWSVAFALSVVGLLITIVNFAFCQRWVKQYGSKPDFEPINYRNLLLTIIGVVALIAIATWLLHNQEVARMALGVVAFGIVVIFGKEAFAMKGAARRKMIVAFILMLEAIIFFVLYSQMPTSLNFFAIRNVEHSILGLAVEPEQYQALNPFWIIIGSPILAAIYNKMGDTLPMPTKFAIGMVMCSGAFLILPLGAKFASDAGIVSVSWLVASYGLQSIGELMISGLGLAMVAQLVPQRLMGFIMGSWFLTTAGANLIGGYVAGMMAVPDNVTDPLMSLEVYGRVFLQIGVATAVIAVLMLLTAPKLHRMTQDDAADKAAKAAVAAENLYFQ\SHHHHHHDYKDDDDK.

#### MdfA

The mdfA gene (NCBI GenBank accession No. AAC73929.1 for *E*. *coli* K-12 substrain MG1655) was cloned upstream of the TEV cleavage-site sequence (TEVcs) of the pWaldo-GFPe vector^54^ via the XhoI and KpnI restriction sites, allowing expression of the MdfA-(TEVcs)-GFP-His_8_ fusion protein. The cloned vector was a kind gift from Mikio Tanabe from the KEK/High Energy Accelerator Research Organization in Japan.

#### MdfA protein sequence

MQNKLASGARLGRQALLFPLCLVLYEFSTYIGNDMRQPGMLENVEQYQAGIEWVPTSMNAYLAGGMFIQWLLGPLSDRIGRRPVMLAGVVWFIVTCLAILLAQNIEQFTLLRFLHGISLCFIGAVGYDAIQESFEEAVCIKITALMANVALIAPLLGPLVGASWIHVLPWEGMFVLFAALAAISFFGLQRAMPETATRIGEKLSLKELGRDYKLVLKNGRFVAGALALGFLSLPLLAWIAQSPIIIITGEQLSSYEYGLLQVPIFGALIAGNLLLARLTSRRTVRSLIIMGGWPIMIGLLVAAAATVISSHAYLWMTAGLSIYAFGIGLANAGLVRLTLFASVMSKGTVSAAMGMLQMLIFTVGIEISKHAWLNGGNGLFNLFNLVNGILLLSLMVIFLKDKQMGNSHEG.

#### Kv1

The Kv1 gene (NCBI GenBank accession No. PA3789) was cloned into the NdeI - BamHI sites of the pnEK–vH expression vector^55^ as a fusion protein with an N-terminal His_6_-tag.

#### Ij1

The Ij1 gene (NCBI GenBank accession No. CAQ31984.1) was cloned into the NcoI - XhoI sites of the pET28a-TEV-His vector (Novagen) as a fusion protein with a C-terminal His_6_-tag.

#### P2X4

The P2X4 orf was cloned into the EcoRI - BamHI sites of the pOET2 vector (Oxford Expression Technologies).

#### P2X4 protein sequence

MAGCCSALAAFLFEYDTPRIVLIRSRKVGLMNRAVQLLILAYVIGWVFVWEKGYQETDSVVSSVTTKVKGVAVTRTSKLGFRIWDVADYVIPAQEENSLFVMTNVILTMNQTQGLCPEIPDATTVCKSDASCTAGSAGTHSNGVSTGRCVAFNGSVKTCEVAAWCPVEDDTHVPQPAFLKAAERFTLLVKNNIWYPKFNFSKRNILPNITTTYLKSCIYDAKTDPFCPIFRLGKIVENAGHSFQDMAVEGGIMGIQVNWDCNLDRAASLCLPRYSFRRLDTRDVEHNVSPGYNFRFAKYYRDLAGNTQRTLIKAYGIRFDIIVFGKAGKFDIIPTMINIGSGLALLGMATVLCDIIVLYCMKKRLYYREKKYKYVEDYEQGLASELDQGSSGTETSQVAPA.

#### BR protein sequence

mlellptavegvsqaqitgrpewiwlalgtalmglgtlyflvkgmgvsdpdakkfyaittlvpaiaftmylsmllgygltmvpfggeqnpiywaryadwlfttplllldlallvdadqgtilalvgadgimigtglvgaltkvysyrfvwwaistaamlyilyvlffgftskaesmrpevastfkvlrnvtvvlwsaypvvwligsegagivplnietllfmvldvsakv gfglillrsraifgeaeapepsagdgaaatsd.

#### LacY

The LacY gene was cloned into the pWaldo-GFP plasmid (a pET28 derived system) downstream the TEV site as a GFP-his tagged fusion.

#### LacY protein sequence

MYYLKNTNFWMFGLFFFFYFFIMGAYFPFFPIWLHDINHISKSDTGIIFAAISLFSLLFQ PLFGLLSDKLGLRKYLLWIITGMLVMFAPFFIFIFGPLLQYNILVGSIVGGIYLGFCFNA GAPAVEAFIEKVSRRSNFEFGRARMFGCVGWALCASIVGIMFTINNQFVFWLGSGCALILAVLLFFAKTDAPSSATVANAVGANHSAFSLKLALELFRQPKLWFLSLYVIGVSCTYDVFDQQFANFFTSFFATGEQGTRVFGYVTTMGELLNASIMFFAPLIINRIGGKNALLLAGTIMSVRIIGSSFATSALEVVILKTLHMFEVPFLLVGCFKYITSQFEVRFSATIYLVCFCFFKQLAMIFMSVLAGNMYESIGFQGAYLVLGLVALGFTLISVFTLSGPGPLSLLRRQVNEVA.

#### Im1

The Im1 gene (NCBI GenBank accession No. EF1051) was cloned into a pET28-derived vector with a C-terminal His_8_-tag.

#### Protein expression

The **DgoT**, **DtpA** and **MdfA** proteins were expressed in *E*. *coli* C41(DE3) cells. For protein expression, cells were grown in terrific broth (TB) media supplemented with 30 μg/ml kanamycin. Cultures were grown at 37 °C and protein expression was induced with 0.2 mM IPTG at an OD_600nm_ of 0.6–0.8. After induction, culture growth continued at 18 °C for 16–18 hours. Cells were harvested by centrifugation (9379 rcf, 15 minutes, 4 °C using a JLA 8.1 rotor in an Avanti JXN-26 centrifuge, Beckman Coulter), and the pellet was stored at −20 °C until further use. Typically, around 20 g of biomass (wet weight) per litre of culture were obtained.

The **Kv1** protein was expressed in *E*. *coli* C43(DE3) cells. Cultures were grown in TB media at 37 °C until an OD_600nm_ of 2, cooled down to 20 °C and induced with 0.1 mM IPTG. After induction, culture growth continued at 20 °C for over-night expression. Cells were harvested by centrifuging (4000 rcf, 25 min, 6 °C) and the pellet was stored at −20 °C until further usage.

The **Ij1** protein was expressed in *E*. *coli* LEMO21(DE3) cells^56^. Cultures were grown in ZY media with 0.3 mM rhamnose at 37 °C until an OD_600nm_ of 1, cooled down to 20 °C and induced with 0.1 mM IPTG. After induction, culture growth continued at 20 °C for over-night expression. Cells were harvested by centrifuging (5000 rcf, 25 min, 6 °C) and the pellet was stored at −20 °C until further usage.

The **P2X4** protein was expressed in *Trichoplusia ni* (T.ni) insect cells. Cells with expressed Rho-tagged P2X4 were obtained from Cube Biotech GmbH.

The **LacY** and **Im1** proteins were expressed in *E*. *coli* C43(DE3) cells in LB media; 20 mL of inoculation culture were added to 1 L flask LB media with kanamycin and incubated at 37 °C, shaking at 260 rpm. When the OD_600nm_ reached 0.5 the temperature was reduced to 25 °C and induced with 0.4 mM IPTG. The culture was left growing for further 16 hours.

### Protein purification

#### DgoT, DtpA and MdfA

Purification was carried out similarly as described for other transporters^33–35^. The cell pellet was resuspended in lysis buffer (20 mM NaP at pH 7.5, 300 mM NaCl, 5% (v/v) glycerol, 15 mM imidazole, 5 ml of lysis buffer per gram of wet weight pellet), supplemented with lysozyme (1 mg/ml final concentration), DNase (5 units/ml) and 0.5 mM tris(2-carboxyethyl)phosphine (TCEP). The cells were lysed by three cycles using an EmulsiFlex-C3 (Avestin) at 10.000–15.000 psi. Recovered material was centrifuged to remove non-lysed cells (9379 rcf, 15 minutes, 4 °C using a JLA 8.1 rotor in an Avanti JXN-26 centrifuge, Beckman Coulter and the supernatant was subjected to ultracentrifugation to collect the total membrane fraction (95834 rcf, 1 hour, 4 °C using a 45 Ti rotor in an Optima XE-90 centrifuge, Beckman Coulter). Membranes were resuspended in lysis buffer supplemented with protease inhibitors (one tablet per 100 ml lysis buffer, Roche), and 0.5 mM tris(2-carboxyethyl)phosphine (TCEP), and solubilized by adding 1% n-dodecyl-β-D-maltoside (DDM) detergent. Solubilized protein was first purified by immobilized-metal affinity chromatography (IMAC) on a gravity column. The beads were pre-equilibrated in Wash 1 buffer (20 mM HEPES pH 7.5, 300 mM NaCl, 5% (v/v) glycerol, 15 mM imidazole 0.5 mM TCEP, 0.03% DDM) and incubated with the solubilized membrane proteins for one hour at 4 °C on a rotating wheel. Loaded beads were extensively washed with wash buffer with increasing imidazole concentrations (20 mM HEPES at pH 7.5, 300 mM NaCl, 5% glycerol, 15–40 mM imidazole, 0.5 mM TCEP, 0.03% DDM). The protein was eluted from the column with a buffer containing high imidazole concentration (20 mM HEPES at pH 7.5, 150 mM NaCl, 5% glycerol, 300 mM imidazole, 0.5 mM TCEP, 0.03% DDM) and combined with 1 ml of TEV protease at 1 mg/ml to cleave the His-tag during dialysis overnight at 4 °C. Typically, 1 mg of TEV protease was sufficient to cleave the tag from the purified protein from 3 liters of culture. The dialysis buffer contained 20 mM HEPES at pH 7.5, 150 mM NaCl, 5% glycerol, 0.5 mM TCEP, 0.03% DDM. Cleaved protein was recovered by negative IMAC. A second purification step was done by size-exclusion chromatography (SEC). The cleaved protein was concentrated to 5 ml using a 100 kDa concentrator (Corning® Spin-X® UF concentrators) and run on an ÄKTA Pure system (GE Healthcare Life Sciences), using a HiLoad 16/600 Superdex 200 (S200) column (GE Healthcare Life Sciences) in SEC buffer (20 mM HEPES at pH 7.5, 150 mM NaCl, 5% glycerol, 0.5 mM TCEP, 0.03% DDM). Fractions containing the protein were pooled and concentrated to 5 mg/ml, flash frozen and stored at −80 °C until further use.

#### Kv1

The cell pellet was resuspended in lysis buffer (30 mM TrisHCL pH 7.5; 300 mM NaCl; 10% glycerol, 2 mM MgCl_2_, 2 µg/ml DNase I; 200 µg/ml lysozyme). The cells were lysed by passing three times through an EmulsiFlex-C3 (Avestin); the lysate was centrifuged (25000 × g; 30 min; 6 °C) supernatant was taken and spun again at 150000 × g; 1 h; 6 °C. The membrane pellets were collected and resuspended in high salt buffer (30 mM TrisHCl pH 7.5 800 mM NaCl; 10% glycerol) and ultracentrifuged (150000 rcf; 1:35 h; 6 °C). Membrane pellets were resuspended in 30 mM TrisHCl pH 7.5; 300 mM NaCl, 10% glycerol, flash frozen in liquid nitrogen and stored at −20 °C. Protein was solubilized by adding 1% DDM and followed by centrifugation at 50000 rcf for 30 min at 6 °C. Solubilized protein was first purified by a nickel-IMAC beads in a gravity column; washed with 10 mM and 30 mM imidazole in 30 mM TrisHCl pH 7.5; 300 mM NaCl, 10% glycerol; 0.03% DDM, and eluted with 250 mM imidazole. Elution fractions containing the protein were concentrated and loaded onto a gel filtration 10/300 S200 column in 20 mM HEPES pH 7.4, 150 mM NaCl and 0.03% DDM. Fractions containing the protein (5 ml) were concentrated in an Amicon® Ultra 4 ml centrifugal filter devices (50 kDa MWCO) to 0.5 ml, final concentration of 9 mg/ml and stored at 4 °C.

#### Ij1

The cell pellet was resuspended in lysis buffer (30 mM TrisHCL pH 7.5; 200 mM NaCl; 5% glycerol, 2 mM MgCl_2_, 2 µg/mL DNase I; 200 µg/ml lysozyme). The cells were lysed by passing three times through an EmulsiFlex-C3 (Avestin); the lysate was centrifuged (25000 × g; 30 min; 6 °C) supernatant was taken and spun again at 150000 × g; 1 h; 6 °C. The membrane pellets resuspended in buffer (30 mM TrisHCl pH 7.5; 200 mM NaCl, 5% glycerol), flash frozen in liquid nitrogen and stored at −20 °C. Protein was solubilized by adding 1% DDM and followed by centrifugation at 50000 rcf for 30 min at 6 °C. Solubilized protein was first purified by nickel-IMAC in a gravity column; washed with 40 mM imidazole in 30 mM TrisHCl pH 7.5; 200 mM NaCl; 5% glycerol; 0.03% DDM and eluted with 300 mM imidazole. Eluted fractions were combined with 1 mg of TEV protease/mg of protein to perform the His-tag cleavage during dialysis overnight at 4 °C. Protein solution was added to gravity nickel-NTA column to perform a reverse IMAC; the flow-through was collected, concentrated and loaded onto a gel filtration 10/300 S200 column in 20 mM HEPES pH 7.4, 150 mM NaCl and 0.03% DDM. Fractions containing the protein were concentrated in an Amicon® Ultra 4 ml centrifugal filter devices (100 kDa MWCO) to a final concentration of 2.5 mg/ml and stored at 4 °C.

#### P2X4

Protein was purified using PureCube Rho1D4 MagBeads following manufacturer’s protocol. Membrane solubilization was achieved by using 2% DDM. Elution fractions were concentrated and loaded onto a gel filtration HiLoad 16/600 Superdex 200 pg column in SEC buffer (150 mM NaCl, 50 mM Tris, 0.05% (w/v) DDM, 0,005% (w/v) CHS Anatrace (CAS# 102601-49-0), 5% v/v glycerol, pH 7). Fractions containing protein (6 ml) were pooled and concentrated in a Millipore device (30 kD MWCO) to 250 μl, final concentration of 0.95 mg/ml and flash frozen using liquid nitrogen. Note that the theoretical MW for P2X4 is around 44.5 kDa and the other bands correspond to monomer/dimer/trimer on the SDS PAGE (see Supplementary Fig. 1).

#### LacY

The resuspended membranes were solubilized in 1x PBS, 150 mM NaCl, 1% DDM and incubated at 4 °C for 2 hours, followed by centrifugation at 100,000 rcf at 4 °C for 45 min. The supernatant was mixed with Ni-NTA (IMAC) resin pre-equilibrated in x1 PBS, 150 mM NaCl, 0.1% DDM and 10 mM imidazole. The protein was eluted with an elution buffer containing a final concentration of 250 mM imidazole. After the first IMAC, the eluted protein was dialyzed overnight at 4 °C against a large volume of buffer (20 mM Tris-HCl, pH 7.5, 150 mM NaCl, 0.03% DDM). An equimolar amount of TEV protease was added for a complete overnight digest at 4 °C. The dialyzed fraction was submitted to a reverse-IMAC to remove the His-tagged TEV protease, cleaved GFP-His_8_-tag and co-eluting contaminating proteins. A final SEC was run in 20 mM Tris-HCl, pH 7.5, 150 mM NaCl, 0.03% DDM. The protein was concentrated using a 100-kDa centrifugal concentrator (Vivaspin) in 20 mM Tris-HCl pH 7.5, 150 mM NaCl, 0.03% DDM and stored at −80 °C (after snap-freeze in liquid nitrogen) at 10 mg/ml. Note that the theoretical MW for LacY is around 47 kDa but this construct runs on the SDS gels between 38 and 28 kDa. Two bands are always visible with the upper band corresponding to the dimer (see Supplementary Fig. 1).

#### Im1

The resuspended membranes were solubilized in 1x PBS, 250 mM NaCl, 1% DDM and incubated at 4 °C for 2 hours, followed by centrifugation at 100,000 rcf at 4 °C for 45 min. The supernatant was mixed with Ni-NTA (IMAC) resin in x1 PBS, 250 mM NaCl, 0.2% DDM and 10 mM imidazole. The protein was eluted with an elution buffer containing a final concentration of 250 mM imidazole. A final SEC was run in 20 mM Tris-HCl pH 7.5, 300 mM NaCl, 0.02% DDM. The protein was concentrated using a 100-kDa centrifugal concentrator (Vivaspin) in 20 mM Tris-HCl pH 7.5, 300 mM NaCl, 0.02% DDM and stored at −80 °C (after snap-freeze in liquid nitrogen) at 14 mg/ml.

#### Detergent exchange

Purified DtpA (in 0.03% DDM) was loaded onto SD200 Increase 10/300 GL gel filtration column in the following SEC buffer: 20 mM HEPES 7.5, 50 mM NaCl, 5% glycerol, 0.01% LMNG, 0.5 mM TCEP.

#### Screen sample preparation

Each condition from the detergent screen “Analytic Selector Kit” (Anatrace, product number AL-SEL) (12.5 μl) was pipetted into a PCR plate and mixed with 10 μl of 2x protein buffer (without any detergent and glycerol). Protein stock (2.5 μl) was added to obtain a final protein concentration in the range of 0.1–0.5 mg/ml and thoroughly mixed by pipetting. The plate was briefly spun down in a swing-bucket centrifuge and incubated for 1 hour at room temperature prior to the thermal denaturation assay. Fluorescence and scattering of detergents without proteins were measured in 50 mM Tris, pH 7.5 and 200 mM NaCl (see Supplementary Table 2).

#### Thermal denaturation assay

Each sample was used to fill two standard grade NanoDSF capillaries (Nanotemper) and loaded into a Prometheus NT.48 device (Nanotemper) controlled by PR.ThermControl (version 2.1.2). Excitation power was pre-adjusted to get fluorescence readings above 2000 RFU for F330 and F350, and samples were heated from 20 °C to 90 °C with a slope of 1 °C/min. An XLSX file with “processed data” was exported from PR. ThermControl and used for further analysis.

#### Data analysis

The fluorescent readouts used in the screening (F330, F350 and F350/F330 Ratio) are redundant, and in most cases analyzing the Ratio data was sufficient. In case of LacY F330 was chosen based on the higher signal strength. In case of BR an unfolding transition was observed in all readouts, however, the Ratio data appeared noisier and F330 was used instead.

A two-state unfolding model is only applicable for reversible unfolding events not compatible with large molecular weight IMPs. Therefore the fitted parameters described below as T_m_, T_agg_ and T_onset_ are only apparent rather than absolute thermodynamic parameters. Raw curves were fit to the equation described by Santoro and Bolen^56^ with Gibbs free energy expressed as a function of temperature^57^, and protein heat capacity change assumed to be zero:

$${\rm{\Delta }}{\rm{G}}({\rm{T}})={\rm{\Delta }}\mathrm{Hm}\ast (1-{\rm{T}}/{\rm{Tm}}),$$

where ΔG is the Gibbs free energy of unfolding, ΔHm is the apparent enthalpy of unfolding at Tm and T_m_ is the melting temperature. T_onset_U_ was calculated from ΔHm as follows:

$${{\rm{T}}}_{\mathrm{onset}\_{\rm{U}}}=1/(1/{\rm{Tm}}+{\rm{R}}\ast \,\mathrm{ln}(0.01/0.99)/{\rm{\Delta }}\mathrm{Hm}),$$

where R is the universal gas constant.

We decided to compare samples in terms of T_m_ and T_onset_ rather than ΔHm and T_m_, because in the former case both curve characteristics have the same dimensionality (temperature degrees), while ΔHm is in J/mol.

For scattering curves the Gibbs free energy function was substituted to a descriptive equation, which represents the transition with two values: T_agg_ – aggregation temperature (50% molecules aggregated) and Tonset – onset temperature (1% molecules aggregated):

$${\rm{Agg}}({\rm{T}})=({\rm{T}}-{{\rm{T}}}_{{\rm{agg}}})\ast \,\mathrm{ln}(0.01/0.99)/({{\rm{T}}}_{\mathrm{onset}\_\mathrm{Agg}}-{{\rm{T}}}_{{\rm{agg}}})$$

Covariance matrix of the fit parameters was used to calculate standard deviation of T_m_ and T_agg_ of individual samples and if the value exceeded 0.5 K the sample was discarded. Fit parameters for the duplicates were averaged to obtain standard deviation.

Derivative curves were obtained from experimental curves by applying a Savtizky-Golay filter with 4-order polynomial and 10 °C window size.

For correlation analysis between micelle size and T_m_ or T_agg_ we chose Spearman’s rank correlation coefficient (ρ). This coefficient quantifies how well the relationship between two can be described by a monotonic function, but does not make any assumptions about the function itself. Computation was performed using stats.spearmanr function from scipy^57^.

#### Replicates

The standard deviation for the calculated T_m_ and T_agg_ for six replicate experiments is usually below 0.3 °C due to the low intrinsic error of the nanoDSF measurements and the accuracy of replicates of dilution. As demonstrated by Malo *et al*.^58^, measuring in a duplicate reduces the imprecision by 29%. In our experience working with an n = 2 produces reliable and consistent results and would be a good compromise when dealing with scarce material.

#### Software

Data processing and visualization was done in MoltenProt^59^, which is written in Python^60^ using the following modules: scipy^57^, numpy^61^, pandas^62^, matplotlib^63^.

#### Chemicals

- Selector detergent screen (Anatrace). See Table 1 and Supplementary Information.
- Bacteriorhodopsin (25 mg/ml in 250 mM Na/K phosphate buffer, pH 6.5, 1% OG) was obtained from Cube Biotech.

## Supplementary information

Suplementary information

## Data Availability

The datasets and analysis generated in the current study are available upon request (m.garcia@embl-hamburg.de).
