# Supplementary material for: High-throughput stability screening for detergent-solubilized membrane proteins
Source: Sci Rep. 2019 Jul 17;9:10379. doi: 10.1038/s41598-019-46686-8 (PMC6637136; doi:10.1038/s41598-019-46686-8)
Supplement: Supplementary file 1 — Suplementary information [file 41598_2019_46686_MOESM1_ESM.pdf]

Supplementary Information

High-throughput stability screening for detergent-solubilized membrane proteins

Vadim Kotov[1,2,3], Kim Bartels[1,4], Katharina Veith[5], Inokentijs Josts[5], Udaya K. Tiruttani Subhramanyam[1,6], Christian Günther[4], Jörg Labahn[1,6], Thomas C. Marlovits[1,2,3], Isabel Moraes[7,8], Henning Tidow[5], Christian Löw[1,4,9] and Maria M. Garcia-Alai[4*].

1. Centre for Structural Systems Biology, Notkestrasse 85, D-22607 Hamburg, Germany (CSSB).
2. University Medical Center Hamburg-Eppendorf (UKE), Institute for Structural and Systems Biology, Notkestrasse 85, D-22607 Hamburg, Germany.
3. German Electron Synchrotron Centre (DESY), Notkestrasse 85, D-22607 Hamburg, Germany.
4. European Molecular Biology Laboratory Hamburg, Notkestrasse 85, D-22607 Hamburg, Germany.
5. The Hamburg Centre for Ultrafast Imaging (CUI) and Department of Chemistry, University of Hamburg, Martin-Luther-King-Platz 6, D-20146 Hamburg, Germany.
6. Research Centre Jülich, Institute of Complex Systems (ICS-6), Wilhelm-Johnen-Straße, D-52425, Juelich, Germany.
7. National Physical Laboratory, Hampton Road, Teddington TW11 0LW, UK.
8. Research Complex at Harwell, Rutherford Appleton Laboratory, Harwell Science
9. Department of Medical Biochemistry and Biophysics, Karolinska Institutet, Scheeles väg 2, SE-17177 Stockholm, Sweden.

Corresponding author (*): m.garciaalai@embl-hamburg.de

Supplementary table 1

Protein name	DDM in original SEC buffer	Readout used in analysis
DtpA	0.03%	Ratio
DgoT	0.03%	Ratio
LacY	0.03%	F330
Kv1	>0.03%	Ratio
Ij1	0.03%	Ratio
Im1	0.02%	Ratio
MdfA	0.03%	Ratio
P2X4	>0.05% (CHS 0.005%)	Ratio
BR	1% OG	F330

Table S1: Concentration of DDM in SEC buffer. The percent of DDM used for each example protein during purification varies from 0.02% to 0.05% (w/v). The amount of DDM used during the SEC step has been empirically determined for each protein to retrieve well behaved samples (see Supplementary Fig.1).

Supplementary table 2

Detergent	T_{agg} (ºC)
C9_5.9 mM Anapoe®-20 (Tween 20) (T-20)	87.8
C11_11.5 mM Anapoe-X-100 (TX-100)	70.0
C12_10 mM Anapoe-X-114 (TX-114)	39.5
D3_15 mM [Octylphenoxy]Polyethoxyethanol (NID-P40)	60.5
D10_46 mM Triethylene Glycol Monohexyl Ether (C6E3)	50.4
D11_60 mM Tetraethylene Glycol Monohexyl Ether (C6E4)	68.5
D12_ mM Pentaethylene Glycol Monohexyl Ether (C6E5)	74.0
E1_42 mM Pentaethylene Glycol Monoheptyl Ether (C7E5)	67.3
E2_20 mM Tetraethylene Glycol Monooctyl Ether (C8E4)	36.3
E3_17.75 mM Pentaethylene Glycol Monooctyl Ether (C8E5)	78.5
E4_25 mM Hexaethylene Glycol Monooctyl Ether (C8E6)	71.3
E5_8.1 mM Pentaethylene Glycol Monodecyl Ether (C10E5)	41.0
E6_9 mM Hexaethylene Glycol Monodecyl Ether (C10E6)	59.9
E7_3.9 mM Polyoxyethylene(9)decyl Ether (C10E9)	87.9
E8_6.9 mM Heptaethylene Glycol Monododecyl Ether (C12E7)	65.1
E9_9 mM Octaethylene Glycol Monododecyl Ether (C12E8)	75.1
E10_5 mM Polyoxyethylene(9)dodecyl Ether (C12E9)	80.0
E11_10 mM Polyoxyethylene(10)dodecyl Ether (C12E10)	86.0
E12_10 mM Polyoxyethylene(8)tridecyl Ether (C13E8)	60.5

Table S2: Detergents that display scattering transitions in the absence of protein.

Figure legends:

Figure S1. a) SDS PAGE for all purified proteins used in the assay. M lane shows molecular weight markers in kDa. Note: A figure with the full SDS-PAGEs for each protein can be found on Figure S9. b) SEC profiles for all purified proteins in the assay with exception of BR which was purchased from Cube biotech. SEC conditions are described in Material and Methods.

Figure S2. Thermal denaturation of IMPs. a) Surface representations for Halobacterium salinarum Bacteriorhodopsin (BR) PDB:4XXJ, peptide transporter DtpA (DtpA) PDB: 6GS1, multidrug transporter MdfA (MdfA) PDB:4ZP0, human P2X4 ion channel (P2X4) based on PDB:4DW1 and lactose permease (LacY) PDB:1PV6. Tryptophan residues exposed to the surface are highlighted in pink. b) Example of a "Z thermal denaturation curve" and the visualization of Tm at the inflection point and the change in the slope reporting Tonset_U.

Figure S3. Thermal denaturation curves following the intrinsic fluorescence of the Trp residues (raw data) for all nine proteins included in the study at the starting condition (A2). We have used the fluorescence ratio (F350/F330) for fitting T_m for all proteins with the exception of Lac Y and BR where the fluorescence at 330 nm has been used. For these two cases we have included the F350/F330 graphs as insets. In the case of LacY the ratio of the single wavelengths mutually cancels out the transition. For BR, the single wavelength (330nm) scan displays a sharper transition than the ratio.

Figure S4.

Detergent equilibration time. 1mg/ml DtpA (a) and 3.4 mg/ml DgoT (b) aliquots have been diluted 1/10 from the starting condition A2 (0.03% DDM (w/v)) to 2 mM LMNG (condition D4 in the screen) and incubated prior to perform DSF runs. DSF measurements were recorded after 10 minutes (pink), 30 minutes (red), 1 hour (green), 2 hours (cyan) and overnight (blue). All curves overlap indicating that the detergent mix is stabilized after 10 minutes of mixing.

Figure S5. Tenfold dilution vs detergent exchange. a) SEC profile for DtpA in 0.01% LMNG w/v (see methods). b) DSF measurement for DtpA purified in 0.03% DDM w/v (A2 condition from the screen) shown in cyan and after SEC detergent exchange to 0.01% LMNG w/v in blue. The green curve corresponds to the ten-fold dilution of the DDM purified protein in the LMNG

D4 condition of the screen. c) 1st Derivative F350/F330 for DtpA vs. Temperature: at the starting condition A2 (0.03% DDM (w/v); cyan), diluted 1/10 to LMNG (1 mM; green) and after SEC detergent exchange in LMNG (blue).

Figure S6. Correlation between protein stability and micelle size. Supplementary figure to Table 4. Apparent T_m or T_{agg} obtained after fitting of DSF and scattering experiments from the detergent screening were plotted as a function of the micellar molecular weight (kDa) of the detergent in that particular condition. Spearman correlation coefficients have been calculated to test if there is a monotonic relationship between the melting or aggregation temperatures and the size of the micelle (Table 4).

Figure S7. Stability analysis for Kv1 and BR. a) Kv1 scatter plot of T_{onset_U} vs T_m: No outliers from the linear trend are observed. b) Heat-map for delta T_m and delta T_{agg} for BR using either OG or DDM as reference. Grey wells indicate samples where the data could not be obtained. Yellow squares correspond to conditions with no difference detected compared to the original condition (OG or DDM) at the beginning of the assay. Red and green wells are colored according to the difference between that condition and position A2 with a difference of +10 °K or above (green) and -10 °K or below (red). T_m is calculated from the fluorescence recorded at 330 nm.

Figure S8. Stability of a misfolded protein. We have used Im1 as an example of a protein that is either not correctly folded after purification in DDM or the used thermostability assay cannot report the unfolding event for unknown reasons. However, we have detected certain conditions that display unfolding transitions after dilution to the detergent screen. a) First derivatives for the thermal denaturation of Im1 in selected detergents. b) DSF heat-map (ratio F350/F330) for the assay for Im1.

Figure S9. Full SDS-PAGEs for all purified proteins used in the assay. Labels indicate molecular weight of the marker lane (in kDa).

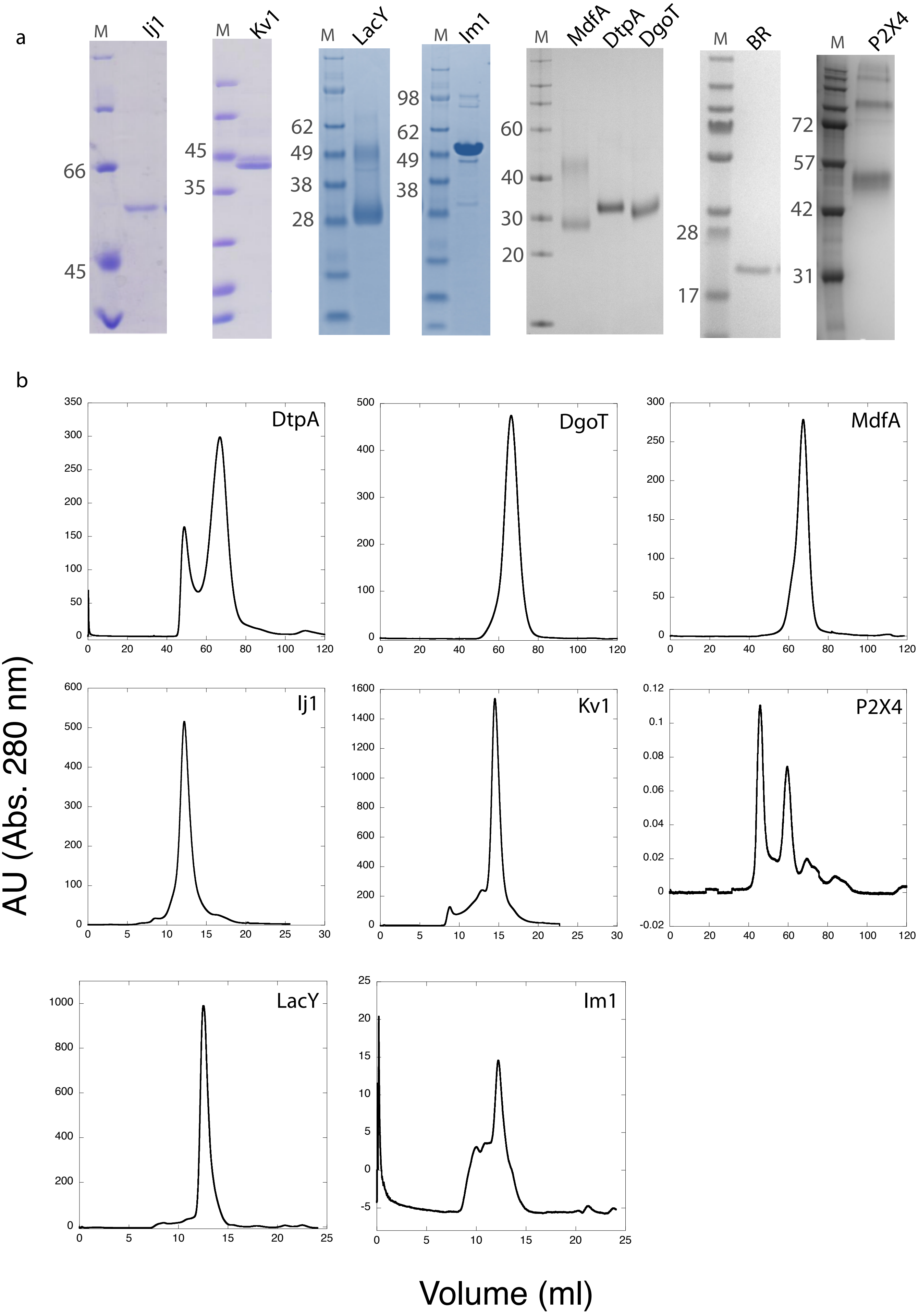

Supplementary Fig. S1

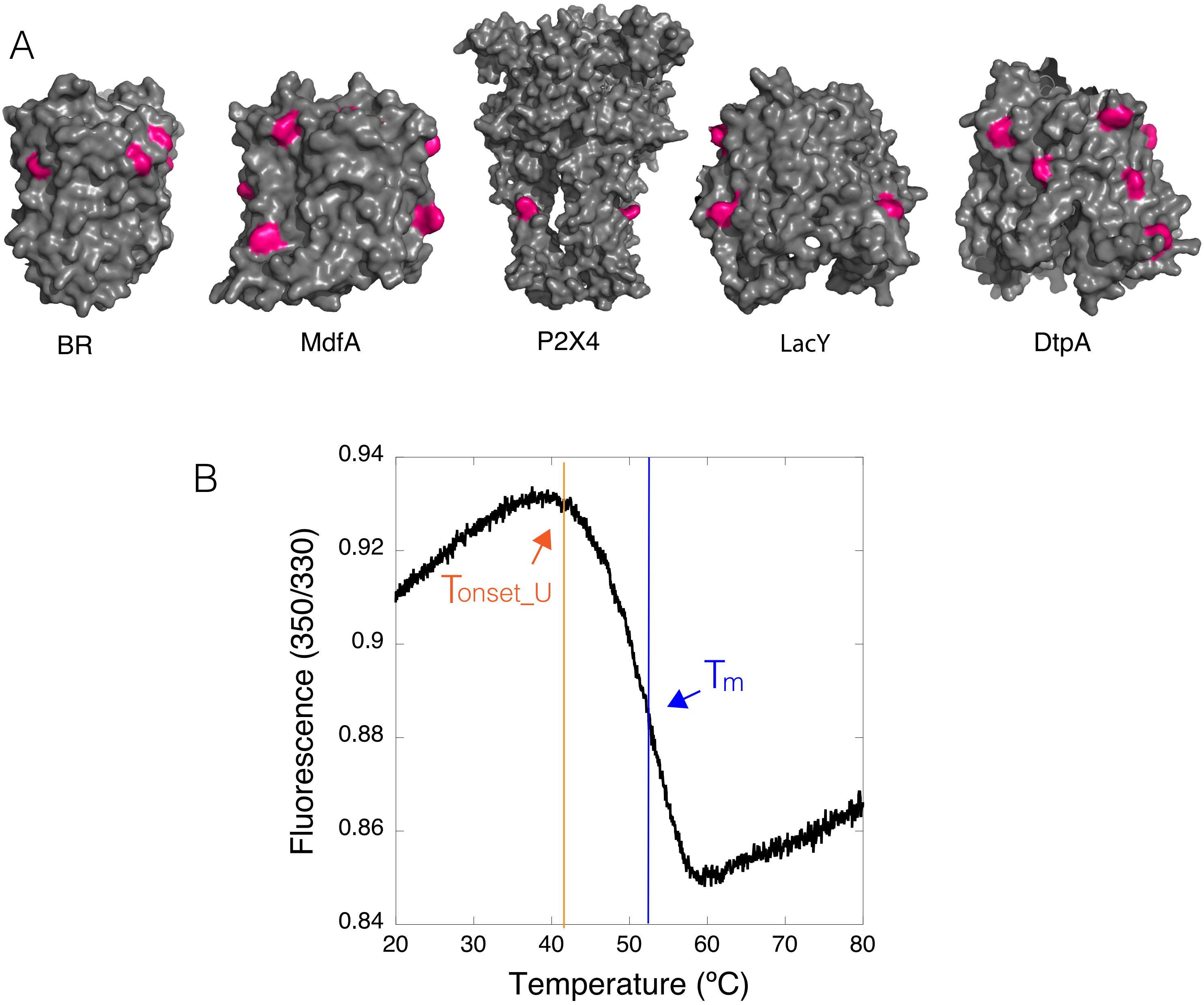

Supplementary Fig. S2

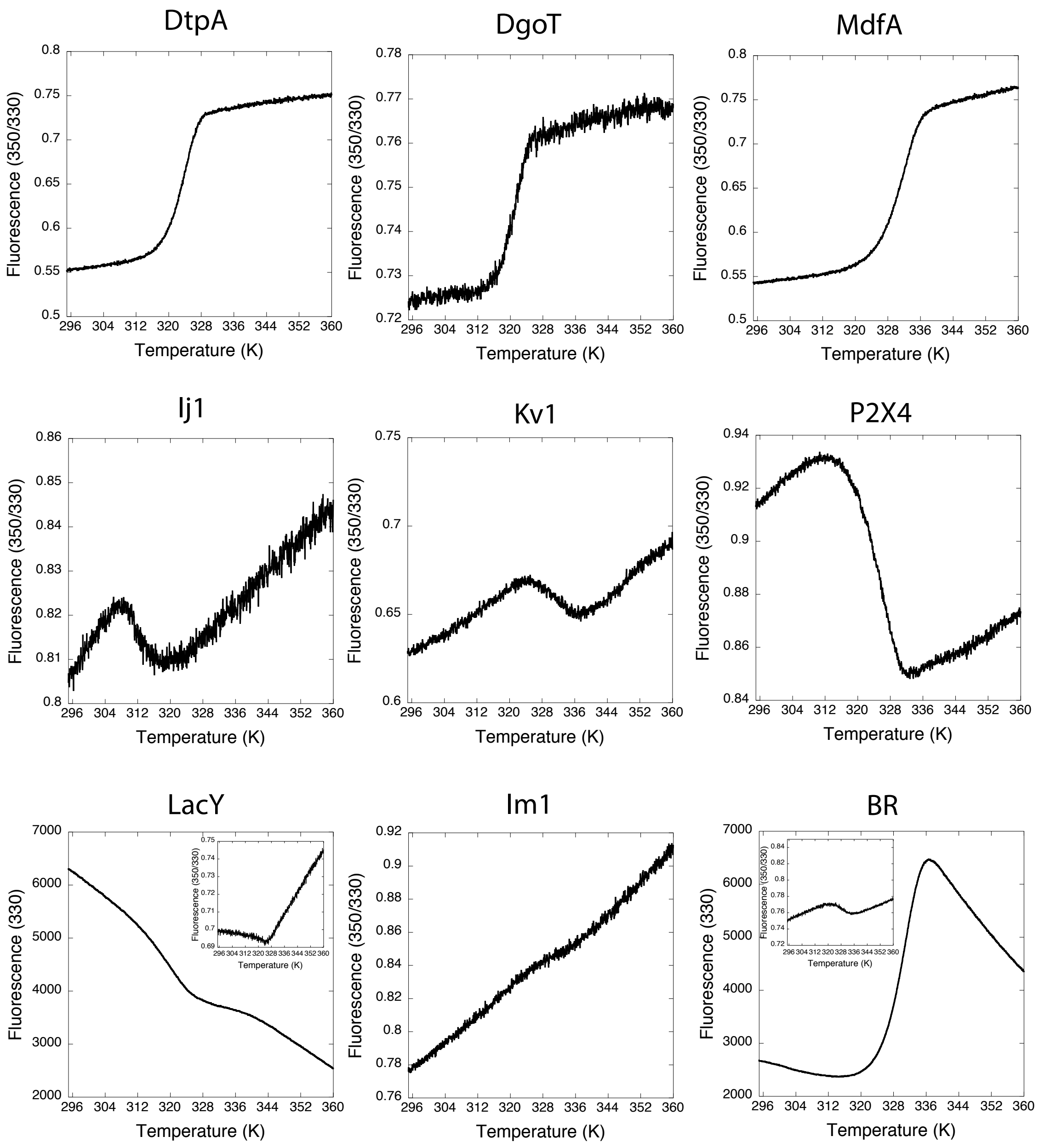

Supplementary Fig. S3

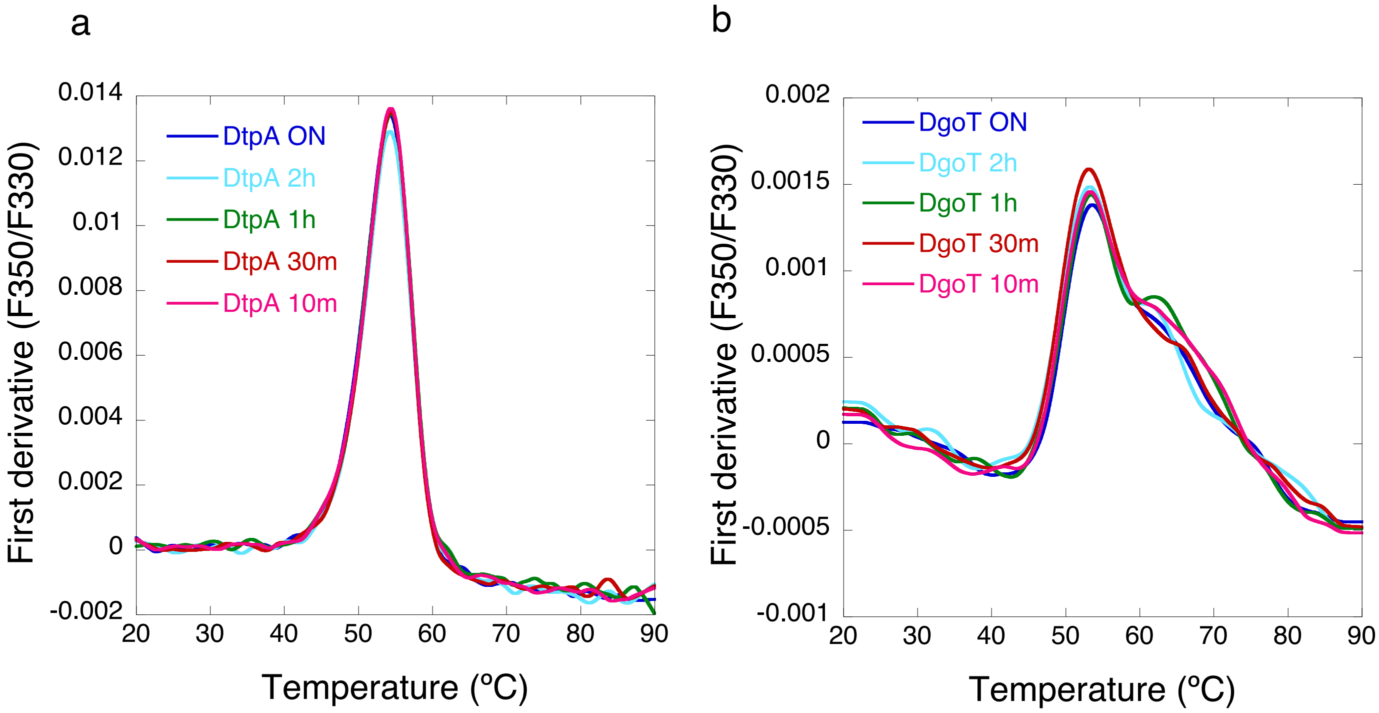

Supplementary Fig. S4

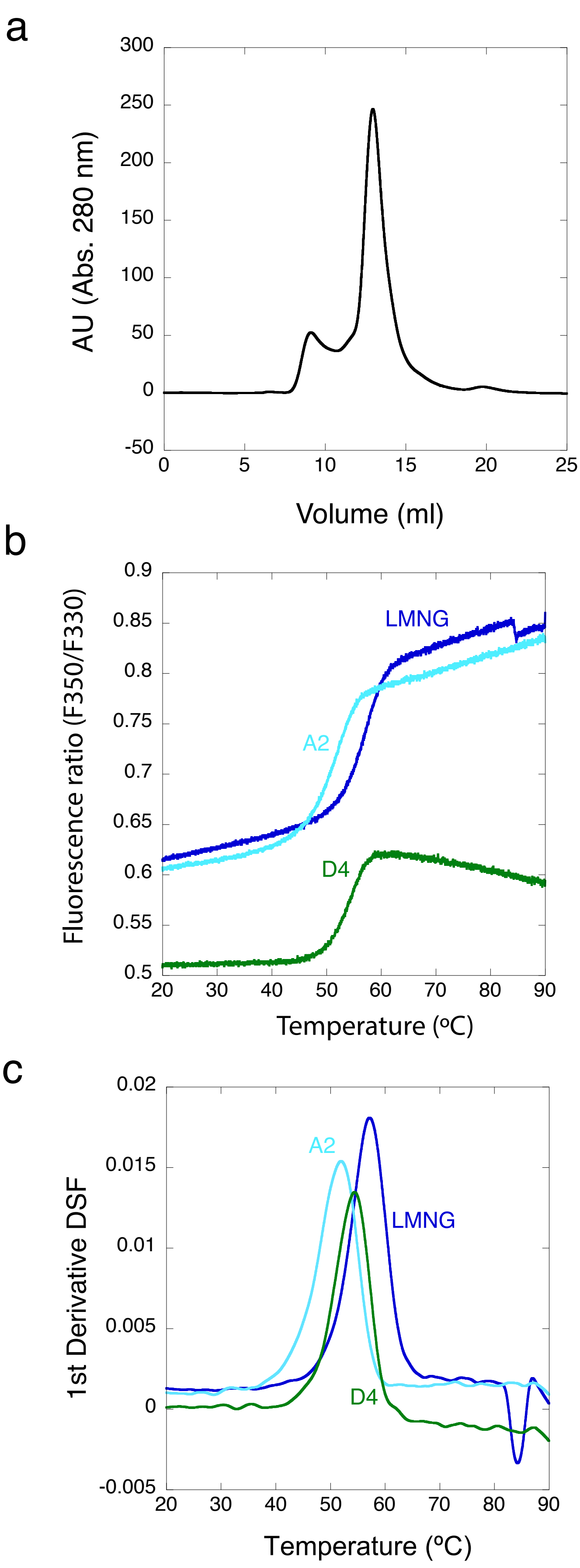

Supplementary Figure 5

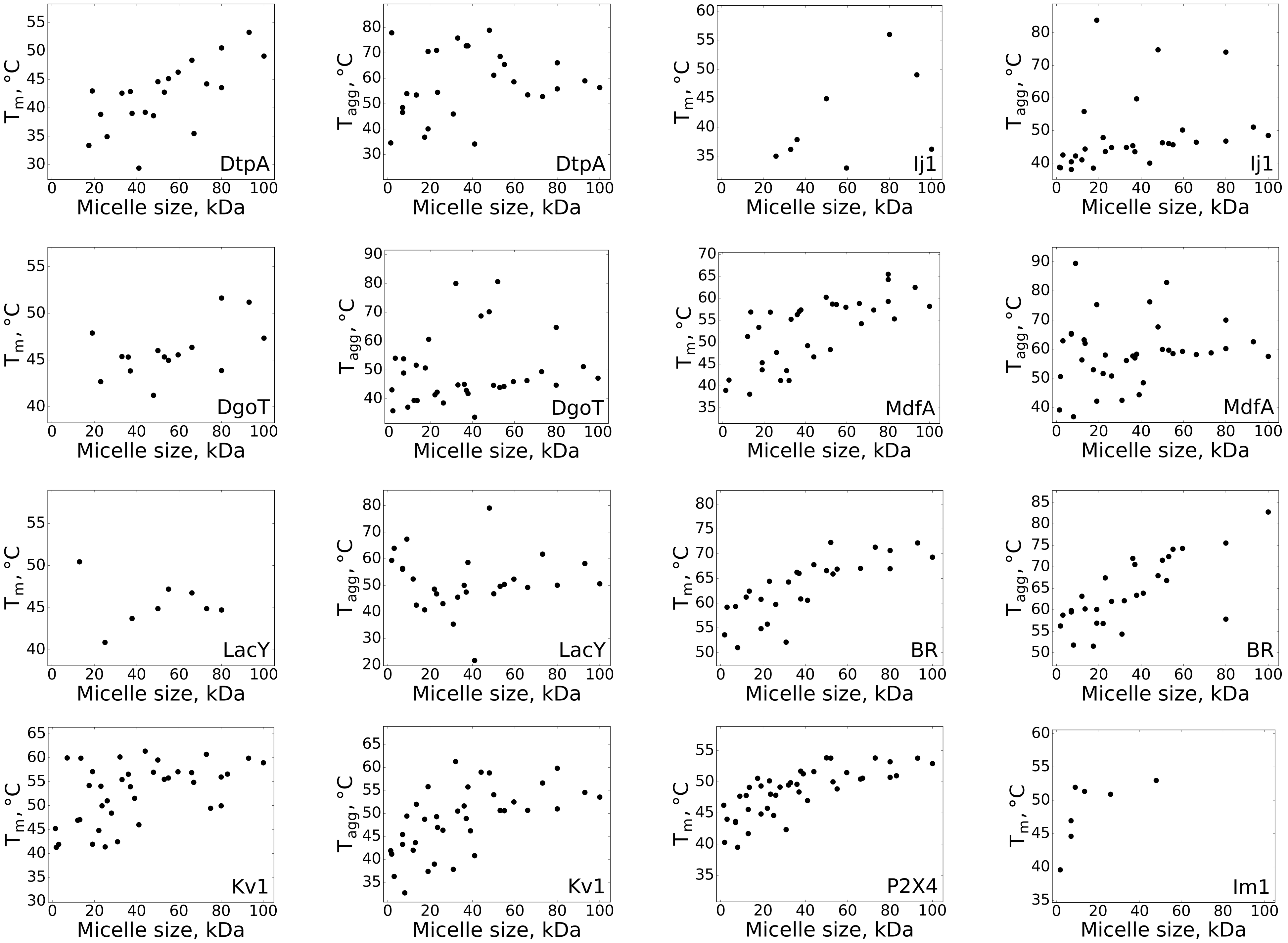

Supplementary Fig. S6

a

b

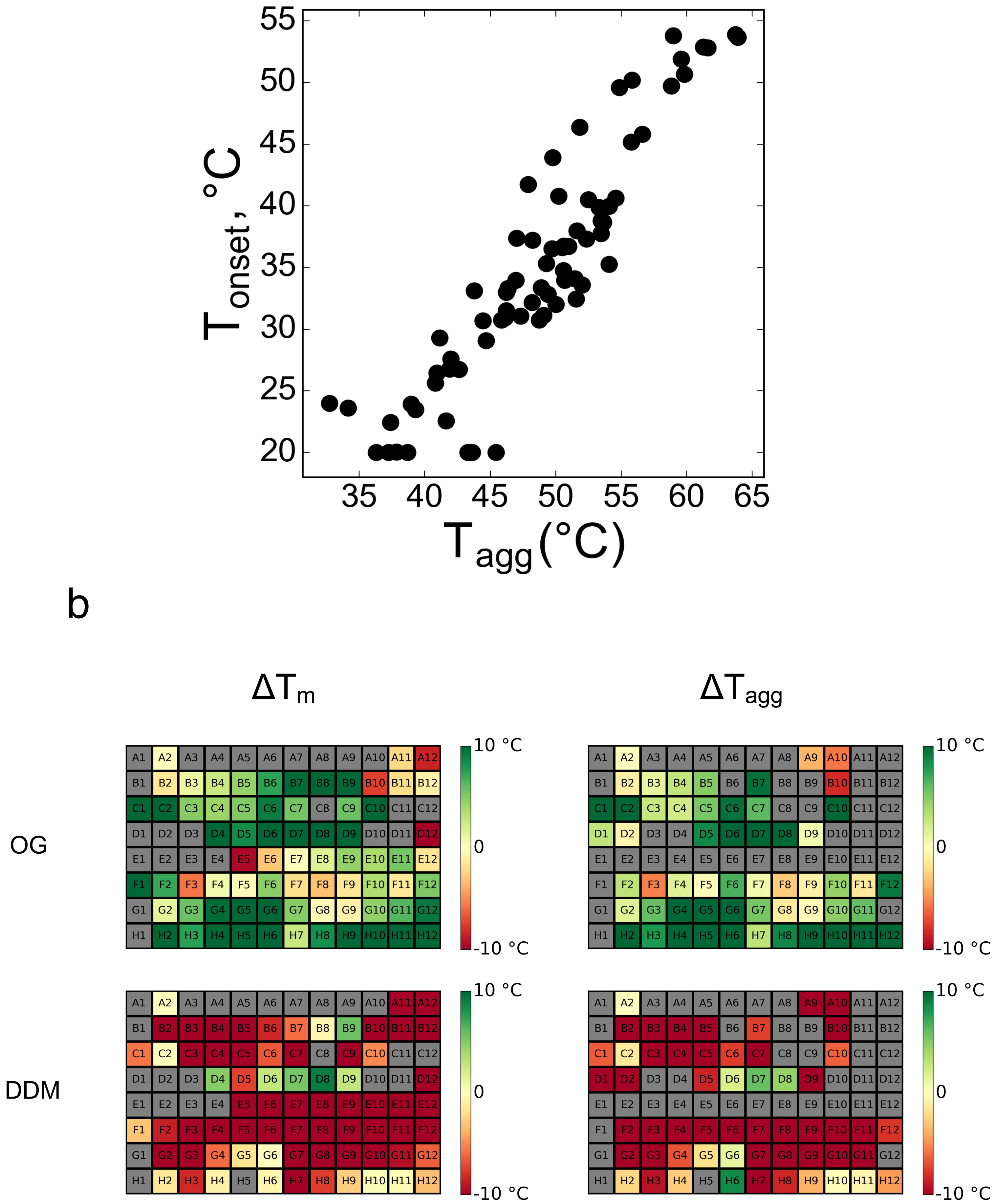

Supplementary Fig. S7

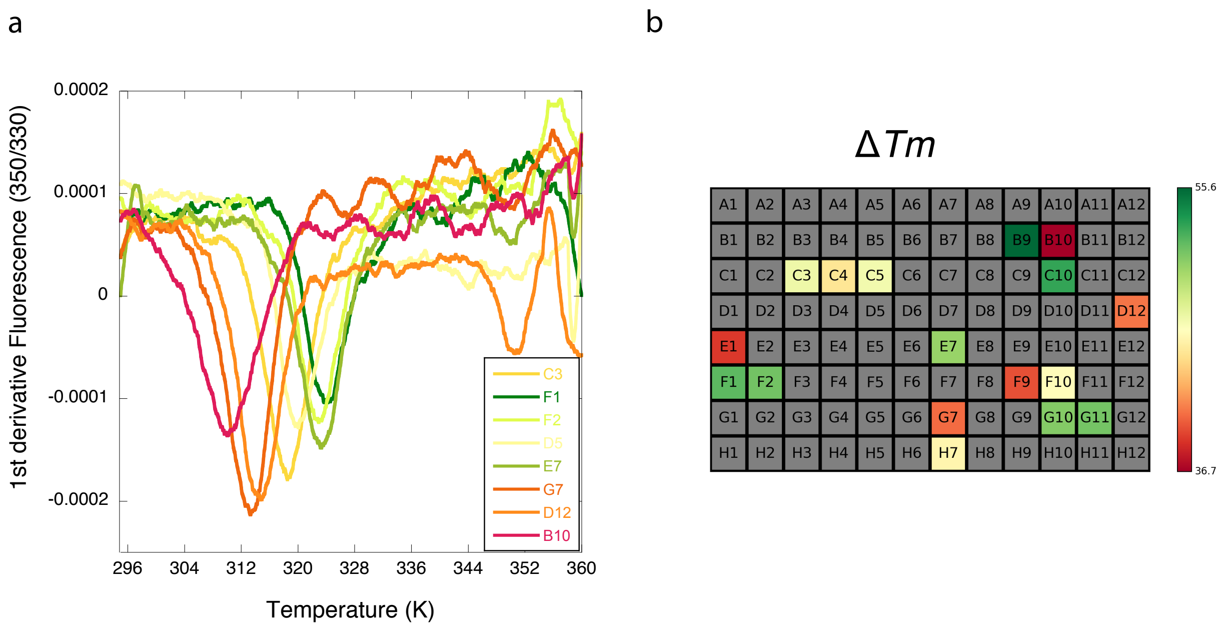

Supplementary Fig. S8

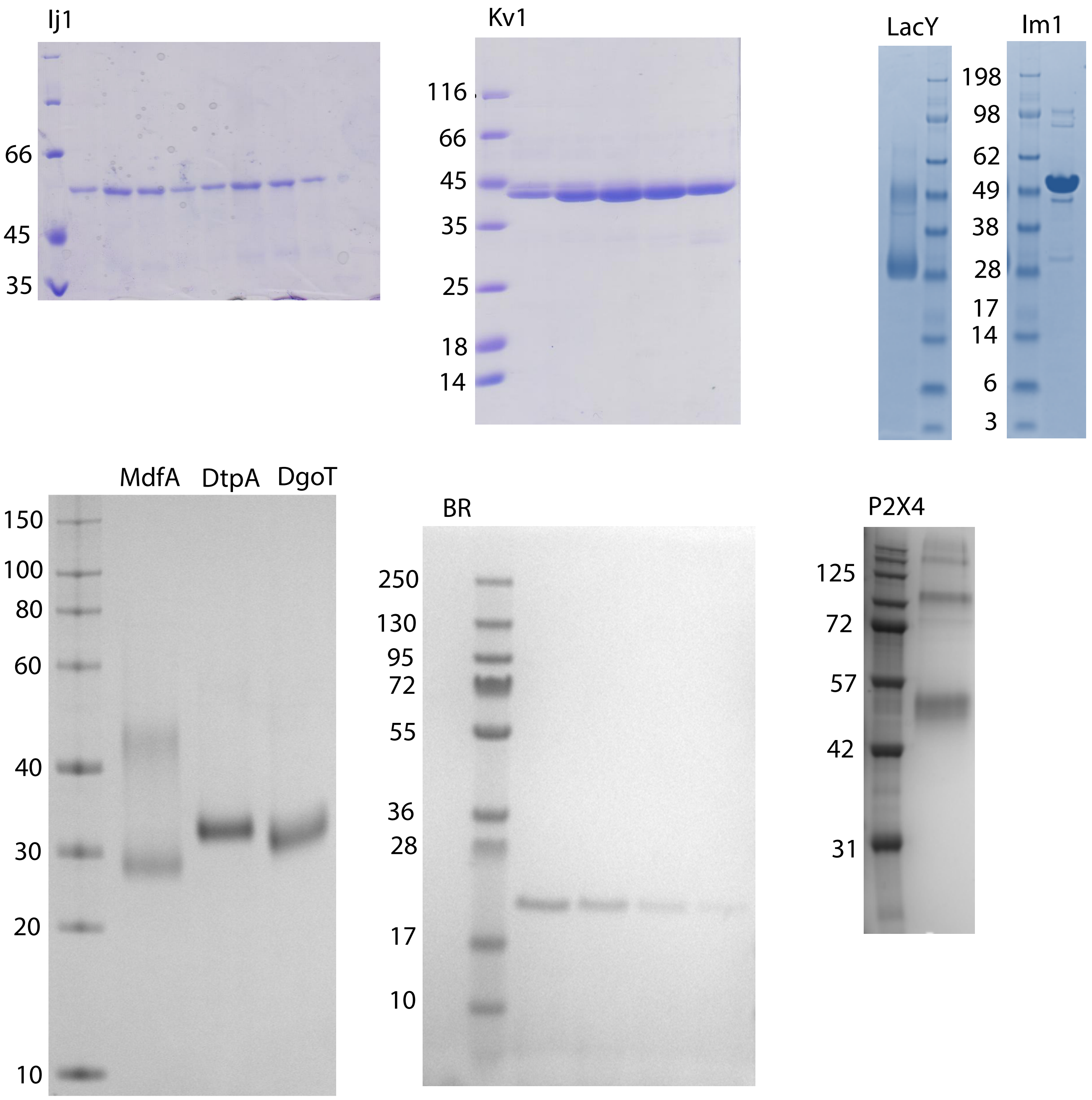

Supplementary Fig. S9
